# Determinants of Sickness Absence and Return to Work Among Employees with Common Mental Disorders: A Scoping Review

**DOI:** 10.1007/s10926-017-9730-1

**Published:** 2017-10-04

**Authors:** Haitze de Vries, Alba Fishta, Beate Weikert, Alejandra Rodriguez Sanchez, Uta Wegewitz

**Affiliations:** 1 Department of Health Sciences, Community and Occupational Medicine, University Medical Center Groningen, University of Groningen, Hanzeplein 1, Postbus 30001, 9700 RB Groningen, The Netherlands; 2 Department of Evidence-based Occupational Health, Workplace Health Management, Federal Institute for Occupational Safety and Health (BAuA), Berlin, Germany

**Keywords:** Common mental disorders, Sickness absence, Return to work, Prognostic factors, Scoping review

## Abstract

*Purpose* To present an overview of the existing evidence on prognostic factors of (recurrent) sickness absence (SA) and return to work (RTW) among workers with a common mental disorder (CMD). This scoping review provides information about determinants for SA and RTW, which could be used to develop better interventions aimed at the prevention of SA and promotion of RTW among workers with a CMD. *Methods* Relevant articles were identified in PubMed, Embase, PsycINFO, PSYNDEX, and SINGLE up to October 2016. In order to be included, studies should provide insight into prognostic factors of SA or RTW of workers with a CMD. We classified all factors according to the domains of the International Classification of Functioning, Disability and Health. *Results* Our searches identified 2447 possible relevant articles, of which 71 were included for data extraction. There is consistent evidence in ≥3 studies that previous episodes of CMD, higher symptom severity, previous absenteeism, co-morbidity, high job demands, low job control, high job strain, female gender, lower educational level, smoking behavior, and low perceived general health are predictors of SA in people with CMDs. Earlier RTW is consistently predicted by lower symptom severity, having no previous absenteeism, younger age, and positive expectations concerning sick-leave duration or RTW. *Conclusions* The amount of research on determinants for SA and RTW in workers with CMD has increased dramatically in recent years, although most studies are from the Netherlands and Scandinavia. There are some research gaps identified in this scoping review that need further attention in primary and secondary studies. Based on the summary of the evidence, we provide guidance for policy, practice and research.

**Electronic supplementary material:**

The online version of this article (doi:10.1007/s10926-017-9730-1) contains supplementary material, which is available to authorized users.

## Introduction

Common mental disorders (CMDs) are long-lasting predictors of onset, duration and recurrence of sickness absence (SA), reduced productivity, work disability, and early retirement [1–3]. In the present study, the definition of CMD included anxiety disorders, depressive disorders, and stress-related disorders (adjustment disorders, burnout). Depression, for example, is estimated to be one of the ten leading contributors to disability in the world [4]. The prevalence of CMD among the general working population during the last 12 months preceding assessment has been estimated to be approximately 17.6% [5].

CMDs generate high direct and indirect costs for society at several levels [6, 7]. These not only have a financial burden on companies and governments, but also affect the wellbeing of individuals, who see their working- and earnings capacity reduced, or can no longer participate in the labor market. Tackling mental ill-health of the working-age population is becoming a key issue for labor market and social policies in OECD countries. Governments increasingly recognize that policy has to play a major role in keeping people with CMDs in employment or bringing those outside of the labor market back to it [8]. Therefore, understanding which factors help or hinder workers’ capacity to stay at work or successful return to work (RTW) when impaired by a CMD, is a relevant public health focus.

Only three systematic reviews have studied the prognostic factors of work outcomes in people among a working age population with mental illness [9–11]. Despite the relatively recent publication dates of these reviews, included studies were relatively outdated and focused not exclusively on CMDs. Although determinants for SA and RTW among people with a CMD have been studied in the past, an overview of these factors is lacking. From existing literature it is known that RTW-interventions for people with a CMD are scarce and that the effectiveness of RTW-interventions for workers with mental health problems is generally poor [12–15]. Clearly, there is a need to develop more adequate interventions to prevent SA and improve RTW for workers with a CMD, and also to carry on studies that investigate its effectiveness. When future interventions are designed based on the known prognostic factors for SA and RTW, their effectiveness can potentially be improved. Hence, there is a need for an overview of determinants for SA and RTW of workers with a CMD, and an indication of which determinants have the strongest prognostic value.

In this article we present a scoping review on the existing latest empirical evidence on the prognostic factors of SA and RTW among workers with a CMD. An overview of determinants for SA and RTW will allow us to report about the factors that have been studied so far, and to identify the omissions in the literature. Differences across countries will be discussed. The relevance of this scoping review was to improve the knowledge for researchers and practitioners on the factors that should be considered in designing better interventions aimed at preventing SA and improving RTW among the working population with a CMD.

## Methods

This study was a scoping review, which uses a strict methodology for collecting, synthesizing, appraising and presenting findings from existing research on a topic [16]. A study protocol was designed a priori [17]. The methodological steps in this scoping review were adapted from Arksey and O’Malley [18]. These can be grouped into a framework of five main stages encompassing the whole process: (i) identifying the research question, (ii) identifying relevant studies, (iii) selecting studies for analysis, (iv) charting the data, and (v) collating, summarizing and reporting the results. Each stage of the research process is comprehensively described below.

### Identifying the Research Question

The definition of the research question entailed a preliminary phase in which a broad set of questions were posed. The methodology of scoping reviews allows a post hoc narrowing of the research question and adoption of the criteria set a priori. Ultimately, the following research questions were defined:

- Which potential risk factors for (recurrent) SA or RTW in workers with CMD have been studied so far?
- What prognostic factors are related to SA due to CMDs?
- What prognostic factors positively or negatively influence the (successful) RTW among employees with CMDs?
- Which prognostic factors are related to the recurrence of SA due to CMDs?
- Where are the omissions in the current knowledge or evidence?
- Which recommendations can be made according to the results?

### Identifying Relevant Studies

Relevant articles were identified by means of a computerized search up to 24 October 2016 in the bibliographic databases PubMed, Embase, PsycINFO, and PSYNDEX, which was followed by a manual search and a search for grey literature. The search strategy was initially formulated for PubMed and was adapted for use in the other databases. Controlled vocabulary search terms (MeSH terms, Emtree terms, PsycINFO and PSYNDEX Descriptors) and free text words were used. Three main terms about prognostic factors, SA and RTW, and CMD were combined with the Boolean operator ‘AND’ to identify studies (exact search strategy available upon request). In order to be included, studies should provide insight into determinants of (long-term) SA or RTW in workers with a CMD. We included systematic reviews of qualitative studies, prognostic studies, and primary studies (e.g. cross-sectional studies, cohort studies, case-control studies and qualitative studies). Narrative reviews, letters, editorials, commentaries, government reports, meeting abstracts, animal or human experimental studies, intervention studies (controlled and uncontrolled studies) were excluded. Additionally, we complemented the database search by a hand search of citations from 3 relevant systematic reviews retrieved by a systematic search in PubMed, EMBASE, PsycInfo and PSYNDEX [9–11], the reference lists of included primary studies, and a search for grey literature in the System for Information on Grey Literature in Europe (SINGLE: http://www.opengrey.eu). We also contacted experts in the field of mental disorders and occupational medicine for relevant studies.

### Selecting Studies for Analysis

Two authors (AF/BW and ARS) independently screened the studies identified in each database on title and abstract. After this first selection, BW/ARS and HdV independently assessed the corresponding full versions of the articles to determine which articles should be included in the full review. Studies were excluded when both reviewers considered it not fulfilling the inclusion criteria. Discrepancies were solved by discussion; when needed a third reviewer (AF) was enrolled. The criteria for inclusion were developed in accordance to the PEO format for observational studies, where population (P) terms were combined with exposure (E) terms and outcomes (O) [19]. For an article to be included in this scoping review, it had to comply with the following three criteria:

1. *Population* Working-age population with a CMD, such as depressive disorders (ICD-10: F32-F34), anxiety disorders (ICD-10 diagnostic categories F40-F42), stress-related disorders, including adjustment disorders (ICD-10: F43) and somatoform disorders (ICD-10: F45), and burnout (ICD-10: Z73.0), but without severe mental disorders (schizophrenia, personality disorders, mental retardation, etc.). In the case that workers with other conditions were among the study population, it was necessary that a separate analysis was performed among the workers with a CMD. Cases where CMD was a comorbid condition, were also excluded. When more than 80 percent of the sample in a study had CMDs, the study was eligible for inclusion.
2. *Exposure* Studies evaluating the exposure to risk factors, or prognostic factors were included. When the research focus was only considering the CMD condition itself as prognostic factor, then the article was excluded.
3. *Outcome* SA, RTW or recurrent SA. The search included other outcomes, such as work ability, work satisfaction and work functioning, but ultimately in this scoping review we focused only on SA and RTW. Articles with the outcomes unemployment, work disability (not defined in terms of SA), work ability, work functioning, and (early) retirement were excluded.

Additionally, only studies published in English, German or Spanish were included.

### Charting the Data

The relevant data for answering our research questions were summarized in a data extraction form by one of the authors (ARS). The accuracy of the extracted information was then corroborated by two other authors (HdV and AF), and improved or complemented when necessary.

We presented the data in chronological order of the outcomes SA, RTW, and recurrent SA. SA should be measured as the number of days or spells of absenteeism within a predetermined time frame, as a percentage in a predefined period, or as currently being absent or not. RTW should be related to an endpoint at which RTW is determined. We considered cessation of disability payments as an acceptable proxy for RTW. Comparison of studies using different RTW definitions appears valid as long as RTW status is not considered as a measure of functional status [20]. Recurrent SA always takes place after a period of RTW, and was defined as having recurrent SA at follow-up “yes” versus “no”, or defined as “time until recurrent SA”. The data extraction form included these main characteristics of the studies: authors, year of publication, geographic location of the study, type of study, time to follow-up, aim of the study, study population (general working population, specific occupational groups, patients with a mental disorder), prognostic factors under study, outcome measures used (definition or operationalization such as duration of SA, SA rates, time until RTW, RTW-rates, etc.), and the associations with corresponding confidence intervals (the maximal adjustment for confounders was chosen). These results are presented in Supplemental Table 1.

### Collating, Summarizing and Reporting the Results

In this stage, we created an overview of all information relevant to answer our research questions. The characteristics of the included primary studies were numerically described, and thematically reported with referral to the research questions [21]. We classified all factors according to the domains of the International Classification of Functioning, Disability and Health (ICF) model, proposed by Heerkens et al.: disease related factors, body functions and structures, activity limitations, participation restrictions, environmental (work related) factors, and personal (work related) factors [22]. The ICF offers a valuable approach to understanding the contextual influences on employee mental health and work disability [23].

A detailed description of the features of the included primary studies allowed us to identify existent research gaps with respect to prognostic factors, outcome and study type. Based on the summary of this evidence, we discuss implications for policy, practice and research [21]. Additionally, in light of the gaps in research identified here, we were able to more clearly state what should be the scope of future systematic reviews or primary studies focusing on the topic of SA or RTW for workers with a CMD.

## Results

**Fig. 1:**
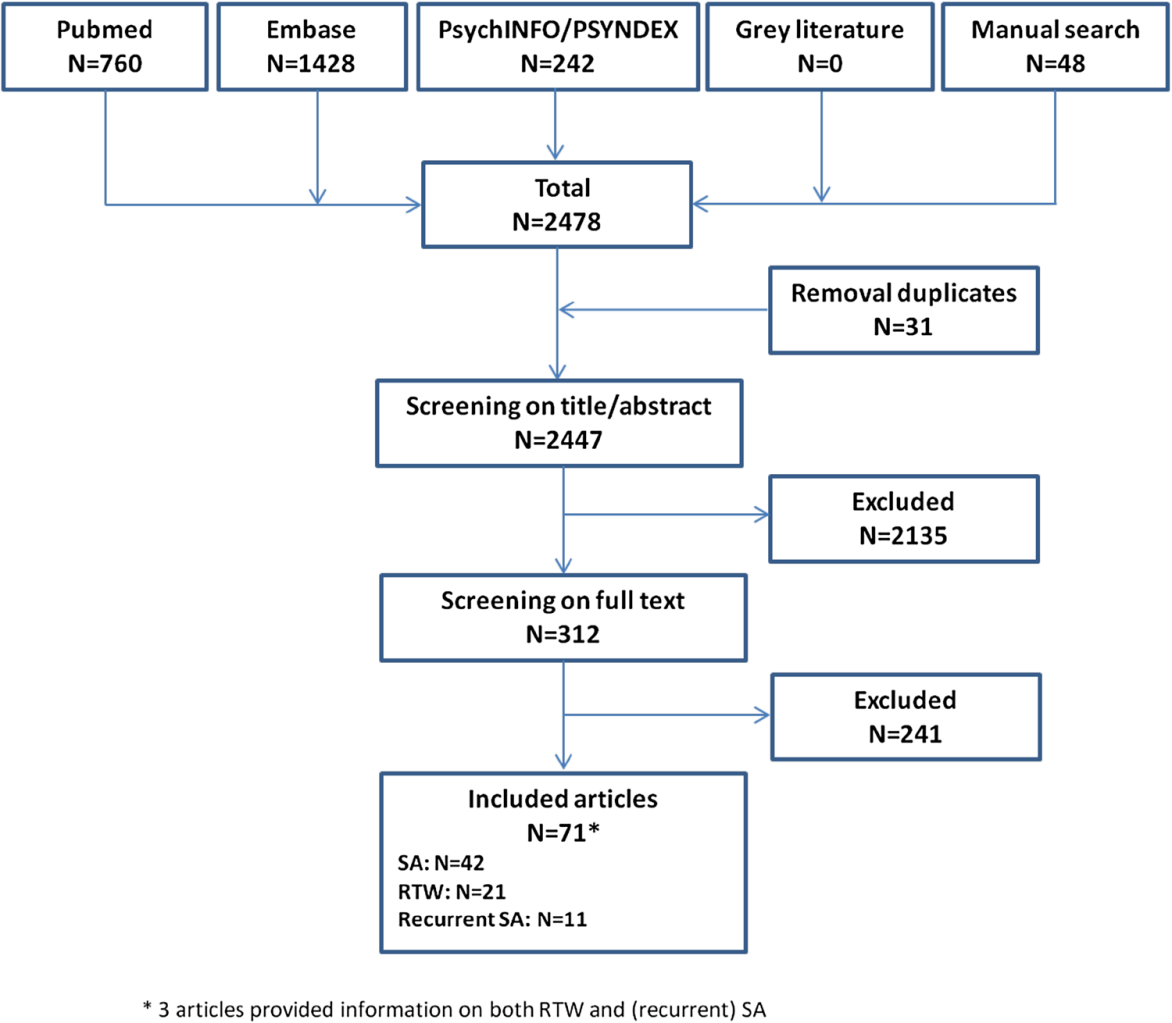
Flow chart of search results and screening stages

Figure 1 shows a flow chart with the results of the search process, in which the number of articles included in the scoping review are presented. Our searches identified 2478 articles. After removal of duplicates, a total of 2447 articles were screened for eligibility, of which 2135 were excluded because the inclusion criteria were not met. The hand search did add 48 articles to the results. After full-text screening of 312 articles, a total of 71 articles from 53 separate studies were deemed relevant and included for analysis. Three articles reported on both SA or RTW and recurrent SA.

Articles that did not fulfill the predefined inclusion criteria were excluded. The reasons for exclusion after screening on title and abstract were because the study population, exposure or outcome were not relevant for this scoping review (children or adolescent, studies of disorders not meeting our CMD definition or only physical conditions, genetic conditions, etc.). At full-text screening, articles were excluded because no information on a CMD (sub)group was provided, no SA- or RTW related outcome was studied, or only diagnose itself was reported as prognostic factor. Study design (methodological papers dealing with specific methods to study RTW, randomized controlled trials and validation studies) was another reason for exclusion. Also language was a reason for exclusion. The reasons for exclusion in both steps were documented in all cases and can be provided upon request.

### General Description of Articles

In Table 1 the general characteristics of the included articles are presented, categorized according to their main outcomes SA (n = 42), RTW (n = 21), and recurrent SA (n = 11). For SA, a total of 78 factors were studied and considered as potential predictors, for RTW 53 factors, and for recurrent SA 24 factors. Most studies were conducted after 2011, in earlier years studies on prognostic factors for SA or RTW in CMD were scarce. Although the studies were carried out in several countries, the predominant amount of studies was from the Netherlands and the Scandinavian countries. Just a few studies were included from the United States and no studies from Germany. Most studies were longitudinal cohort studies (N = 65), although in 6 studies a cross-sectional design was used, with a retrospective data collection on previous treatment, SA, or potential confounders. In 25 of all included studies, the cohort was a general working population, where workers with and without a CMD were compared. In the other 46 studies, a specific CMD population was analyzed longitudinally on SA and RTW outcomes.

**Table 1:** Characteristics of included articles (n = 71)

	Sickness absence (N = 42)		RTW (N = 21)		Recurrent sickness absence (N = 11)	
	N	%	N	%	N	%
Year of publication						
>2013	11	26	4	19	7	64
2010–2013	18	43	10	48	4	36
2006–2009	7	17	3	14		
2002–2005	3	7	3	14		
<2002	3	7	1	5		
Country						
Australia	2	5	1	5		
Belgium	2	5				
Denmark	5	12	4	19		
Finland	6	14	1	5	2	18
France	1	2.3	1	5		
Great Britain	1	2.3				
Japan					2	18
Norway	2	5				
Spain	4	9	1	5	1	9
Sweden	5	12	2	9		
Canada	1	2.3	1	5		
United States	2	5	1	5		
The Netherlands	11	26	9	43	6	55
Study design						
Longitudinal cohort study	35	83	21		11	100
Cross-sectional study, with retrospective data collection	7	17				
Duration of study						
>5 years	5	12	1	5	7	64
1–5 years	12	29	10	48	3	27
1 year	15	36	8	38	1	9
6–12 months	6	14	2	9		
Unclear	4	9				
Diagnose CMD group						
Depression/anxiety	8	19	1	5	1	9
(Major) depression only	17	40	3	14	2	18
CMD	14	33	12	57	8	73
Burnout/stress	3	7	4	19		
Unclear			1	5		
Study population						
General working population	21	50	3	14	3	27
Specific CMD population	21	50	18	86	8	73

### Descriptive Numerical Summary

In Tables 2, 3 and 4 we present a descriptive numerical summary of all prognostic factors and their associations with respectively SA, RTW, and recurrent SA as outcome variables. For a complete description of all included studies, we refer to Supplemental Table 1.

**Table 2:** Overview of prognostic factors of SA in workers with common mental disorders

Factor	Result	Evidence	Outcome
Disease related factors			
Duration of illness last 5 years		Insufficient	
van der Werff et al. [38]	No effect		SA last 6 months
Past history of CMD		3+, 3ne	
Rytsala et al. [28]	Positive		SA or at work
Souêtre et al. [24]	Positive		Past history of SA
Hendriks et al. [60]	No effect		No SA, <2 or >2 weeks
Recurrence			
Rytsala et al. [28]	Positive		SA or at work
van der Werff et al. [38]	No effect		SA last 6 months
Hendriks et al. [60]	No effect		No SA, <2 or >2 weeks
Symptom severity		11+	
Rytsala et al. [28]	Positive		SA or at work
Souêtre et al. [24]	Positive		Past history of SA
van der Werff et al. [38]	Positive		SA last 6 months
Verboom et al. [40]	Positive		days SA last 6 months
Lerner et al. [27]	Positive		SA past 2 weeks
Hees et al. [52]	Positive		% SA in last 4 weeks
Hjarsbech et al. [45]	Positive		>3 weeks SA last year
Hjarsbech et al. [51]	Positive		>3 weeks SA last year
Lexis et al. [35]	Positive		Days SA last 10 months
Stansfeld et al. [41]	Positive		Spells of SA 1991–1998
Hallsten et al. [46]	Positive		SA > 60 consecutive days
Body functions and structures			
Agreeableness		2ne	
Vlasveld et al. [53]	No effect		>2 weeks SA last 6 months
Verboom et al. [40]	No effect		Days SA last 6 months
Conscientiousness		1+, 1−	
Vlasveld et al. [53]	Negative		>2 weeks SA last 6 months
Verboom et al. [40]	Positive		Days SA last 6 months
Extraversion		1−, 1ne	
Vlasveld et al. [53]	Negative		>2 weeks SA last 6 months
Verboom et al. [40]	No effect		Days SA last 6 months
Openness		2ne	
Vlasveld et al. [53]	No effect		>2 weeks SA last 6 months
Verboom et al. [40]	No effect		Days SA last 6 months
Neuroticism		2+	
Vlasveld et al. [53]	Positive		>2 weeks SA last 6 months
Verboom et al. [40]	Positive		Days SA last 6 months
Locus of control		Insufficient	
Vlasveld et al. [53]	Negative		>2 weeks SA last 6 months
Sleeping problems		2+	
Lerner et al. [27]	Positive		SA past 2 weeks
Salo et al. [48]	Positive		SA episode > 9 days
Mental distress		2+	
Foss et al. [36]	Positive		>8 weeks SA last 5 years
Roelen et al. [57]	Positive		>3 weeks SA last year
Fatigue severity		Insufficient	
Roelen et al. [56]	Positive ♂ No effect ♀		>3 weeks SA last year
Reduced concentration		Insufficient	
Roelen et al. [56]	Positive ♂ No effect ♀		>3 weeks SA last year
Activities			
Activity limitations at work		Insufficient	
Sanderson et al. [23]	No effect		SA days last 4 weeks
Low level of physical activity		1+, 1ne	
Verboom et al. [40]	Positive		Days SA last 6 months
Mather et al. [63]	No effect		SA spell last 5 years
(Work) participation			
Past history of absenteeism		5+	
Souêtre et al. [24]	Positive		Past history of SA
Hallsten et al. [46]	Positive		SA > 60 consecutive days
Smith et al. [58]	Positive		Previous claim SA
Riihimaki et al. [59]	Positive		Time spent SA last 5 years
Elovainio et al. [50]	Positive		SA > 9 days last year
Environmental factors			
Family history of depression		Insufficient	
Verboom et al. [40]	No effect		Days SA last 6 months
Previous psychiatric treatment		1+, 1ne	
Elovainio et al. [50]	Positive		SA > 9 days last year
Gasse et al. [49]	No effect		SA > 2 weeks
Size social network		Insufficient	
Verboom et al. [40]	No effect		Days SA last 6 months
Social support (partner, friends)		Insufficient	
Verboom et al. [40]	No effect		Days SA last 6 months
Having had social assistance		Insufficient	
Riihimaki et al. [59]	No effect		Time spent SA last 5 years
Treatment condition (psychiatrist and psychologist vs no specialist)		Insufficient	
Catalina-Romero et al. [39]	No effect		SA duration
Environmental work related factors			
Autonomy		Insufficient	
Smith et al. [58]	No effect		Days of full SA last 2 years
Benefit plan features (days between injury date and 1st day of compensation)		Insufficient	
Smith et al. [58]	Positive		Days of full SA last 2 years
Coworker support		2ne	
Munir et al. [44]	No effect		≥3 weeks SA last 2 years
Clumeck et al. [34]	No effect		SA incidence > 28 days
Decision latitude		Insufficient	
Munir et al. [44]	Negative		≥3 weeks SA last 2 years
Effort reward imbalance		2ne	
Janssens et al. [55]	No effect		SA ≥ 15 days last year
Norlund et al. [43]	No effect		Risk of unchanged SA level
Employment type (full-time vs part-time)		Insufficient	
Smith et al. [58]	No effect		Days of full SA last 2 years
Having a non-permanent contract		Insufficient	
Real et al. [64]	Negative		Long-term SA > 60 days
Function with time pressure		Insufficient	
Smith et al. [58]	Positive		Days of full SA last 2 years
Industry-sector		Insufficient	
Smith et al. [58]	No effect		Days of full SA last 2 years
Job control		3−, 2ne	
Virtanen et al. [33]	Negative		SA > 7 days
Clumeck et al. [34]	Negative		SA incidence > 28 days
Norlund et al. [43]	Negative		Risk of unchanged SA level
Janssens et al. [55]	No effect		SA ≥ 15 days last year
Mather et al. [63]	No effect		SA spell last 5 years
Job demands		5+, 5ne	
Virtanen et al. [33]	Positive ♀ No effect ♂		SA > 7 days
Clumeck et al. [34]	Positive ♂ No effect ♀		SA incidence > 28 days
Norlund et al. [43]	No effect		Risk of unchanged SA level
Janssens et al. [55]	No effect		SA ≥ 15 days last year
Hjarsbech et al. [51]	No effect		>3 weeks SA last year
Kivimaki et al. [37]	Positive		SA (yes vs no)
Melchior et al. [31]	Positive		Days of SA
Mather et al. [63]	Positive		SA spell last 5 years
Job strain		3+, 1ne	
Virtanen et al. [33]	Positive		SA > 7 days
Clumeck et al. [34]	Positive		SA incidence > 28 days
Janssens et al. [55]	No effect		SA ≥ 15 days last year
Mather et al. [63]	Positive		SA spell last 5 years
Iso-strain (job strain and low support)		2+, 1ne	
Clumeck et al. [34]	Positive ♂		SA incidence > 28 days
	No effect ♀		
Mather et al. [63]	Positive		SA spell last 5 years
Organizational justice		2−	
Elovainio et al. [50]	Negative		SA > 9 days last year
Hjarsbech et al. [54]	Negative		>3 weeks SA last year
Overtime work > once a month		Insufficient	
Norlund et al. [43]	No effect		Risk of unchanged SA level
Predictability of work		Insufficient	
Hjarsbech et al. [51]	No effect		>3 weeks SA last year
Quality of leadership		1−, 1ne	
Munir et al. [44]	Negative		≥ 3 weeks SA last 2 years
Hjarsbech et al. [51]	No effect		>3 weeks SA last year
Supervisor support		1−, 2ne	
Clumeck et al. [34]	No effect		SA incidence > 28 days
Foss et al. [36]	Negative		>8 weeks SA last 5 years
Janssens et al. [55]	No effect		SA ≥ 15 days last year
Work environment		Insufficient	
Sanderson et al. [23]	No effect		SA in days last 4 weeks
Work pace		Insufficient	
Hjarsbech et al. [51]	No effect		>3 weeks SA last year
Work stress		Insufficient	
Verboom et al. [40]	Negative		Days SA last 6 months
Working with people		Insufficient	
Norlund et al. [43]	No effect		Risk of unchanged SA level
Personal factors			
Older age		6+, 2−, 6ne	
Druss et al. [26]	Negative		Days SA last year
Lerner et al. [27]	No effect		SA past 2 weeks
Rytsala et al. [28]	Positive		SA or at work
Vaez et al. [32]	Negative		Days SA last year
Foss et al. [36]	No effect ♀ Positive ♂		>8 weeks SA last 5 years
van der Werff et al. [38]	No effect		SA last 6 months
Catalina-Romero et al. [39]	Positive		SA duration
Hallsten et al. [46]	No effect		SA > 60 consecutive days
Verboom et al. [40]	No effect		Days SA last 6 months
Catalina-Romero et al. [47]	Positive		SA ≥ 6 months
Gasse et al. [49]	Positive		SA > 2 weeks
Riihimaki et al. [59]	No effect		Time spent SA last 5 years
Real et al. [64]	Positive		Long-term SA > 60 days
Gender (female vs male)		6+, 1−, 8ne	
Laitinen-Krispijn and Bijl [25]	Negative		≥1 spell of SA last year
Lerner et al. [27]	No effect		SA past 2 weeks
Rytsala et al. [28]	Positive		SA or at work
Vaez et al. [32]	No effect		Days SA last year
Clumeck et al. [34]	No effect		SA incidence > 28 days
Catalina-Romero et al. [39]	Positive		SA duration
Foss et al. [36]	Positive		>8 weeks SA last 5 years
van der Werff et al. [38]	No effect		SA last 6 months
Hallsten et al. [46]	Positive		SA > 60 consecutive days
Smith et al. [58]	No effect		Days of full SA last 2 years
Gasse et al. [49]	Positive		SA > 2 weeks
Elovainio et al. [50]	Positive		SA > 9 days last year
Verboom et al. [40]	No effect		Days SA last 6 months
Riihimaki et al. [59]	No effect		Time spent SA last 5 years
Real et al. [64]	No effect		Long-term SA > 60 days
High educational level		4−, 1ne	
Lerner et al. [27]	Negative		SA past 2 weeks
Foss et al. [36]	Negative		>8 weeks SA last 5 years
Catalina-Romero et al. [39]	Negative		SA duration
Verboom et al. [40]	No effect		Days SA last 6 months
Gasse et al. [49]	Negative		SA > 2 weeks
Socio-economic position		1+, 1−, 1ne	
Vaez et al. [32]	No effect		Days SA last year
Virtanen et al. [33]	Positive		SA > 7 days
Elovainio et al. [50]	Negative		SA > 9 days last year
Household income		Insufficient	
Verboom et al. [40]	No effect		Days SA last 6 months
Cohabiting with children		1+, 1ne	
Hallsten et al. [46]	No effect		SA > 60 consecutive days
Gasse et al. [49]	Positive		SA > 2 weeks
Co-morbidity		6+, 3ne	
Druss et al. [26]	Positive		Days SA last year
Buist-Bouwman et al. [29]	Positive		Days SA last year
Verboom et al. [40]	No effect		Days SA last 6 months
Gasse et al. [49]	No effect		SA > 2 weeks
Hallsten et al. [46]	Positive		SA > 60 consecutive days
Catalina-Romero et al. [47]	Positive		SA ≥ 6 months
van der Werff et al. [38]	No effect		SA last 6 months
Hendriks et al. [60]	Positive		No SA, <2 or >2 weeks
Riihimaki et al. [59]	Positive		Time spent SA last 5 years
Adverse life events		Insufficient	
Verboom et al. [40]	No effect		Days SA last 6 months
Childhood trauma		Insufficient	
Verboom et al. [40]	No effect		Days SA last 6 months
Smoking behavior		3+, 2ne	
Elovainio et al. [50]	Positive		SA > 9 days last year
Hallsten et al. [46]	No effect		SA > 60 consecutive days
Foss et al. [36]	Positive ♀ No effect ♂		> 8 weeks SA last 5 years
Mather et al. [63]	Positive		SA spell last 5 years
Alcohol use		Insufficient	
Mather et al. [63]	No effect		SA spell last 5 years
Unhealthy behavior		Insufficient	
Mather et al. [63]	Positive		SA spell last 5 years
Good general health perception		4−	
Lerner et al. [27]	Negative		SA past 2 weeks
Foss et al. [36]	Negative		>8 weeks SA last 5 years
Peterson et al. [42]	Negative		>90 days SA last 3.5 years
Roelen et al. [61]	Negative		SA > 16 consecutive days
SF-12 domains			
Poor physical functioning		Insufficient	
Roelen et al. [61]	No effect		SA > 16 consecutive days
Poor physical role limitations		Insufficient	
Roelen et al. [61]	No effect		SA > 16 consecutive days
Bodily pain		Insufficient	
Roelen et al. [61]	No effect		SA > 16 consecutive days
Poor vitality		Insufficient	
Roelen et al. [61]	Positive		SA > 16 consecutive days
Poor social functioning		Insufficient	
Roelen et al. [61]	Positive		SA > 16 consecutive days
Poor emotional role limitations		Insufficient	
Roelen et al. [61]	Positive		SA > 16 consecutive days
Poor mental health		Insufficient	
Roelen et al. [61]	Positive		SA > 16 consecutive days
Competitiveness		Insufficient	
Moriana and Herruzo [30]	No effect		SA (yes vs no)
Hostility		Insufficient	
Moriana and Herruzo [30]	No effect		SA (yes vs no)
Avoidance behavior		Insufficient	
Hendriks et al. [60]	No effect		No SA, <2 or >2 weeks
Personal work related factors			
Work motivation		Insufficient	
Roelen et al. [56]	Positive ♂ No effect ♀		>3 weeks SA last year
Job satisfaction		Insufficient	
Moriana and Herruzo [30]	Negative		SA (yes vs no)
Bullying		Insufficient	
Janssens et al. [55]	Positive		SA ≥ 15 days last year
Covert coping towards supervisors and coworkers		Insufficient	
Norlund et al. [43]	Negative		Risk of unchanged SA level
Over commitment		Insufficient	
Norlund et al. [43]	Negative		Risk of unchanged SA level
Exhaustion		2+	
Moriana and Herruzo [30]	Positive		SA (yes vs no)
Peterson et al. [42]	Positive		>90 days SA last 3.5 years
Disengagement		Insufficient	
Peterson et al. [42]	No effect		>90 days SA last 3.5 years
White vs blue collar		Insufficient	
Catalina-Romero et al. [47]	Negative		SA ≥ 6 months
Occupational category		1+, 2ne	
Lerner et al. [27]	No effect		SA past 2 weeks
Hallsten et al. [46]	No effect		SA > 60 consecutive days
Real et al. [64]	Positive		Long-term SA > 60 days
Being a shift worker		Insufficient	
Norder et al. [62]	No effect		Temporary SA
Being self-employed		Insufficient	
Real et al. [64]	Positive		Long-term SA > 60 days

+ positive related with SA, − negative related with SA, *ne* not related with SA

**Table 3:** Overview of prognostic factors of RTW in workers with common mental disorders

Factor	Result	Evidence	Outcome
Disease related factors			
Symptom severity		4−, 2ne	
Hees et al. [76]	Negative		Full RTW > 4 weeks
Brouwer et al. [72]	No effect		Time to full RTW
Brouwers et al. [71]	Negative		Full RTW (yes vs no)
Vemer et al. [80]	No effect		Full RTW > 4 weeks
Dewa et al. [67]	Negative		RTW part-time or full-time
Hoedeman et al. [73]	Negative		Time until complete RTW
Duration of illness		Insufficient	
Brouwers et al. [71]	Negative		Full RTW (yes vs no)
Body functions and structures			
Conscientiousness		Insufficient	
Hees et al. [76]	Positive		Full RTW > 4 weeks
Activities			
No factors were studied multivariate			
(Work) participation			
(Duration of) previous absenteeism		4−	
Engstrom and Janson [70]	Negative		RTW after SA > 28 days
Nielsen et al. [74]	Negative		Time to RTW
Brouwers et al. [71]	Negative		Full RTW (yes vs no)
Prang et al. [85]	Negative		Time to sustained RTW > 30 days
Full-time sick leave at baseline		Insufficient	
Netterstrøm et al. [83]	Negative		RTW after 1 year (yes vs no)
Environmental factors			
Benefit plan features			
High deductible		Insufficient	
Salkever et al. [66]	Negative		Time to RTW (claim duration)
Longer preexisting condition exclusion period		Insufficient	
Salkever et al. [66]	Negative		Time to RTW (claim duration)
Having a carve out		Insufficient	
Salkever et al. [66]	Negative		Time to RTW (claim duration)
Mental health benefits and services availability		Insufficient	
Salkever et al. [66]	Positive		Time to RTW (claim duration)
Disability management practices		Insufficient	
Salkever et al. [66]	Positive		Time to RTW (claim duration)
Long term disability policy provisions			
Higher ratio disability benefits to predisability salary		Insufficient	
Salkever et al. [66]	Negative		Time to RTW (claim duration)
Inability to perform own occupation rather than any appropriate occupation		Insufficient	
Salkever et al. [66]	Negative		Time to RTW (claim duration)
Duration to identification of illness by the Occupational Health Service		Insufficient	
Brouwer et al. [72]	No effect		Time to full RTW
General social support		Insufficient	
Brouwer et al. [72]	No effect		Time to full RTW
Consulting a Psychologist or psychiatrist		Insufficient	
Prang et al. [85]	Negative		Time to sustained RTW > 30 days
Treatment condition		2ne	
Brouwers et al. [71]	No effect		Full RTW (yes vs no)
Vemer et al. [80]	No effect		Full RTW > 4 weeks
Environmental work related factors			
Employment type (private or municipal)		1−, 1ne	
Nielsen et al. [77]	Negative		Time to RTW (benefits stopped)
Engstrom and Janson [70]	No effect		RTW after SA > 28 days
Size of workplace small		1−, 1ne	
Nielsen et al. [77]	No effect		Time to RTW (benefits stopped)
Prang et al. [85]	Negative		Time to sustained RTW > 30 days
Work week > 36 h		Insufficient	
Vemer et al. [80]	Negative		Full RTW > 4 weeks
High decision latitude		Insufficient	
Vemer et al. [80]	Negative		Full RTW > 4 weeks
Low decision authority		Insufficient	
Netterstrøm et al. [83]	Negative		RTW after 1 year (yes vs)
Variety in work		Insufficient	
Norder et al. [84]	Positive		Time until full RTW
High job demands		Insufficient	
Netterstrøm et al. [83]	Negative		RTW after 1 year (yes vs no)
Supervisory behavior			
Communication with employee		Insufficient	
Nieuwenhuijsen et al. [68]	Positive		Time to full RTW
Promoting RTW		Insufficient	
Nieuwenhuijsen et al. [68]	No effect		Time to full RTW
Consulting with professionals		Insufficient	
Nieuwenhuijsen et al. [68]	Negative		Time to full RTW
Social support supervisor		2+	
Vemer et al. [80]	Positive		Full RTW > 4 weeks
Social support leader			
Netterstrøm et al. [83]	Positive		RTW after 1 year (yes vs no)
Coworker support		2+	
Vemer et al. [80]	Positive		Full RTW > 4 weeks
Netterstrøm et al. [83]	Positive		RTW after 1 year (yes vs no)
Interactional justice with supervisor		Insufficient	
Ekberg et al. [82]	Negative		RTW < 3 vs 3–12 months
Contact OP in past 4 weeks		Insufficient	
Brouwers et al. [71]	Negative		Full RTW (yes vs no)
Personal factors			
Older age		8−, 4ne	
Hees et al. [76]	No effect		Full RTW > 4 weeks
Salkever et al. [66]	Negative		Time to RTW (claim duration)
Engstrom and Janson [70]	Negative		RTW after SA > 28 days
Dewa et al. [67]	Negative		RTW part-time or full-time
Young and Russel [65]	Negative		RTW > 4 months
Hoedeman et al. [73]	Negative		Time until complete RTW
Nieuwenhuijsen et al. [69]	Negative		Time to full RTW
Brouwer et al. [72]	No effect		Time to full RTW
Vemer et al. [80]	Negative		Full RTW > 4 weeks
Nielsen et al. [74]	No effect		Time to RTW
Nielsen et al. [77]	No effect		Time to RTW (benefits stopped)
Prang et al. [85]	Negative		Time to sustained RTW > 30 days
Female gender		1+, 3−, 6ne	Female gender
Ekberg et al. [82]	No effect		RTW < 3 vs 3–12 months
Engstrom and Janson [70]	Negative		RTW after SA > 28 days
Dewa et al. [67]	No effect		RTW part-time or full-time
Young and Russel [65]	Positive		RTW > 4 months
Brouwer et al. [72]	No effect		Time to full RTW
Vemer et al. [80]	Negative		Full RTW > 4 weeks
Nielsen et al. [74]	No effect		Time to RTW
Nielsen et al. [77]	No effect		Time to RTW (benefits stopped)
Soegaard [79]	No effect		RTW rate
Prang et al. [85]	Negative		Time to sustained RTW > 30 days
Educational level high		1+, 2−, 2ne	Educational level high
Ekberg et al. [82]	Negative		RTW < 3 vs 3–12 months
Hees et al. [76]	No effect		Full RTW > 4 weeks
Nieuwenhuijsen et al. [69]	Negative		Time to full RTW
Brouwer et al. [72]	Positive		Time to full RTW
Nielsen et al. [77]	No effect		Time to RTW (benefits stopped)
Low socio-economic position		Insufficient	
Virtanen et al. [75]	Negative		RTW after SA ≥ 90 days
Living with adult partner (no children)		Insufficient	
Vemer et al. [80]	Negative		Full RTW > 4 weeks
Co-morbidity		2−, 2ne	
Dewa et al. [67]	No effect		RTW part-time or full-time
Hees et al. [76]	Negative		Full RTW > 4 weeks
Engstrom and Janson [70]	Negative		RTW after SA > 28 days
Nielsen et al. [77]	No effect		Time to RTW (benefits stopped)
General health perception		2+, 1ne	
Hees et al. [76]	Positive		Full RTW > 4 weeks
Sampere et al. [78]	No effect		Time to RTW
Nielsen et al. [77]	Positive		Time to RTW (benefits stopped)
Expectations of treatment		Insufficient	
Ekberg et al. [82]	Positive		RTW < 3 vs 3–12 months
Perceived relation between health and job		Insufficient	
Sampere et al. [78]	Negative		Time to RTW
Attributes cause of absenteeism to family problems		Insufficient	
Brouwers et al. [71]	Negative		Full RTW (yes vs no)
Personal work related factors			
Work motivation		1+, 1ne	
Hees et al. [76]	Positive		Full RTW > 4 weeks
Brouwer et al. [72]	No effect		Time to full RTW
Bullying		2−	
Netterstrøm et al. [83]	Negative		RTW after 1 year (yes vs no)
Prang et al. [85]	Negative		Time to sustained RTW > 30 days
Self-efficacy		2+, 3ne	
Willingness to expend effort in completing a behavior			
Brouwer et al. [72]	Positive		Time to full RTW
Willingness to initiate behavior			
Brouwer et al. [72]	No effect		Time to full RTW
Persistence in the face of adversity			
Brouwer et al. [72]	No effect		Time to full RTW
General self-efficacy			
Sampere et al. [78]	No effect		Time to RTW
RTW self-efficacy			
Nieuwenhuijsen et al. [81]	Positive		Time to full RTW
Need for reduced demands at work		Insufficient	
Ekberg et al. [82]	Negative		RTW < 3 vs 3–12 months
Better workability score (WAI)		2+, 1ne	
Ekberg et al. [82]	Positive		RTW < 3 vs 3–12 months
Sampere et al. [78]	No effect		Time to RTW
Netterstrøm et al. [83]	Positive		RTW after 1 year (yes vs no)
Expectations concerning sick leave duration or RTW		4+, 1ne	
Brouwers et al. [71]	Positive		Full RTW (yes vs no)
Nieuwenhuijsen et al. [69]	Positive		Time to full RTW
Sampere et al. [78]	Positive		Time to RTW
Nielsen et al. [74]	Positive		Time to RTW
Nieuwenhuijsen et al. [81]	No effect		Time to full RTW
Job-turnover intentions		Insufficient	
Ekberg et al. [82]	Negative		RTW < 3 vs 3–12 months
White collar vs blue collar		Insufficient	
Soegaard [79]	Positive		RTW rate
Occupational category		2ne	
Engstrom and Janson [70]	No effect		RTW after SA > 28 days
Nielsen et al. [74]	No effect		Time to RTW
Holding a management function		Insufficient	
Vemer et al. [80]	Positive		Full RTW > 4 weeks

*+* positive related with RTW, *−* negative related with RTW, *ne* not related to RTW

**Table 4:** Overview of prognostic factors of recurrent SA in workers with common mental disorders

Factor	Result	Evidence	Outcome
Disease related factors			
No factors were studied			
Body functions and structures			
No factors were studied			
Activities			
No factors were studied			
(Work) participation			
Previous episode(s) of sickness absence		2+	
Sado et al. [91]	Positive		Time to recurrent SA
Koopmans et al. [88]	Positive		Recurrence of SA
Environmental factors			
Medication use		Insufficient	
Arends et al. [90]	No effect		Recurrent SA at 12 months
Environmental work related factors			
Industry/sector		Insufficient	
Koopmans et al. [88]	Positive		Recurrence of SA
Company size > 100		Insufficient	
Arends et al. [90]	Positive		Recurrent SA at 12 months
Supervisor support		Insufficient	
Arends et al. [90]	No effect		Recurrent SA at 12 months
Coworker support		Insufficient	
Arends et al. [90]	No effect		Recurrent SA at 12 months
Conflict with supervisor		Insufficient	
Arends et al. [90]	Positive		Recurrent SA at 12 months
Job demands		Insufficient	
Endo et al. [92]	Positive		Recurrent SA
Job control		Insufficient	
Endo et al. [92]	No effect		Recurrent SA
Type of social security contributions (general scheme vs self-employed)		Insufficient	
Real et al. [64]	No effect		Recurrent SA
Personal factors			
Older age		1+, 3−, 4ne	
Koopmans et al. [86]	Negative ♀ No effect ♂		Recurrence of SA
Koopmans et al. [88]	Negative		Recurrence of SA
Roelen et al. [87]	No effect		Recurrent SA
Sado et al. [91]	Negative		Time to recurrent SA
Endo et al. [92]	No effect		Recurrent SA
Real et al. [64]	No effect		Recurrent SA
Norder et al. [93]	Positive		Recurrent SA episode
Gender		7ne	
Sado et al. [91]	No effect		Time to recurrent SA
Koopmans et al. [86]	No effect		Recurrence of SA
Koopmans et al. [88]	No effect		Recurrence of SA
Roelen et al. [87]	No effect		Recurrence of SA
Norder et al. [93]	No effect		Recurrent SA episode
Endo et al. [92]	No effect		Recurrent SA
Real et al. [64]	No effect		Recurrent SA
Low socio-economic position		1+, 1ne	
Roelen et al. [87]	No effect		Recurrence of SA
Virtanen et al. [75]	Positive		Recurrence of SA ≥ 90 days
Marital status unmarried		1+, 3ne	
Koopmans et al. [88]	Positive ♀ No effect ♂		Recurrence of SA
Roelen et al. [87]	No effect		Recurrence of SA
Norder et al. [93]	No effect		Recurrent SA episode
Cohabiting		2ne	
Arends et al. [90]	No effect		Recurrent SA at 12 months
Living alone			
Endo et al. [92]	No effect		Recurrent SA
Co-morbid conditions		2+, 1−	
Ervasti et al. [89]	Positive		Recurrence of SA
Koopmans et al. [88]	Positive		Recurrence of SA
Arends et al. [90]	Negative		Recurrent SA at 12 months
Personal work related factors			
Higher salary scale		1−, 1ne	
Koopmans et al. [88]	No effect		Recurrence of SA
Roelen et al. [87]	Negative		Recurrence of SA
Full-time vs part-time employed		3ne	
Koopmans et al. [88]	No effect		Recurrence of SA
Roelen et al. [87]	No effect		Recurrence of SA
Norder et al. [93]	No effect		Recurrent SA episode
Being a shift worker (versus day worker)		Insufficient	
Norder et al. [62]	No effect		Recurrent SA episode
Working as a manager		Insufficient	
Endo et al. [92]	No effect		Recurrent SA
Shorter duration of employment (tenure)		2−, 1ne	
Koopmans et al. [88]	Negative ♀ No effect ♂		Recurrence of SA
Roelen et al. [87]	Negative		Recurrence of SA
Time for commute		Insufficient	
Endo et al. [92]	No effect		Recurrent SA
Job title		Insufficient	
Endo et al. [92]	No effect		Recurrent SA

In Koopmans et al., Roelen et al., and Norder et al. recurrent was defined as SA > 28 days after RTW; in Real et al. recurrence was defined as within 180 days after RTW

*+* positive related with recurrent SA, *−* negative related with recurrent SA, *ne* not related with recurrent SA

#### Sickness Absence

In total, 78 factors for SA in CMD were examined in 42 included articles [23–64]. Table 2 provides an overview of the detected prognostic factors for SA in workers with CMDs, categorized according to the domains of the ICF-framework. The direction of the effect of each factor on SA and the outcome is presented. Only multivariate results are presented. In the majority of the studies, SA was defined as an absence-spell (of certain length) during a certain time span (follow-up period). More detailed information on study objectives, study population, and association estimates with 95% confidence intervals is provided in Supplemental Table 1.

Predictors for SA were observed in all domains of the ICF-framework, except in the ‘activities’ domain. There is consistent evidence from three or more studies that previous episodes of CMD, higher symptom severity (depression, anxiety, burnout), a past history of absenteeism, co-morbidity, high job demands, low job control, high job strain, female gender, lower educational level, smoking behavior, and low perceived general health are predictors of SA in people with CMDs. In addition, there is consistent evidence from two studies that sleeping problems, mental distress, exhaustion, iso-strain (high strain combined with low support), and lower organizational justice are predictors of SA. Consistent evidence for *‘no effect’* was observed for agreeableness, openness, coworker support and effort-reward imbalance. The evidence on age and socio-economic position as predictors for RTW was inconsistent. Because several factors had been studied in only one study, the evidence was qualified as insufficient.

Most robust and modifiable factors, and therefore suitable to be used for interventions to prevent SA, are symptom severity (a positive relation between higher symptom severity and SA was reported in all 11 studies which focused on that factor), job demand and control, job strain, organizational justice, sleeping problems, smoking behavior, and perceived general health. Especially when high perceived job demands are combined with low control and when high strain jobs are combined with low support, there is a higher risk of SA [34, 63]. Higher perceptions of organizational justice were associated with 20–34% lower odds of SA due to CMDs [50]. In another study, organizational justice was only associated with SA for men [54].

#### Return to Work

In total, 53 predictive factors for RTW after SA in people with CMDs were examined in 21 included articles [65–85]. Table 3 provides an overview of the detected prognostic factors for RTW in workers with CMDs. In the majority of the studies, RTW was defined as time to (full) RTW. In Supplemental Table 1, a more detailed overview of the articles is provided.

In all domains of the ICF-framework predictors for RTW were observed, except in the ‘activities’ domain. There is consistent evidence from three or more studies that lower symptom severity, having no previous absenteeism, younger age, and positive expectations concerning sick-leave duration or RTW are predictors of (earlier) RTW in people with CMDs.

There is evidence from two studies that support from supervisor and coworkers, presence of co-morbidity, bullying, (work)self-efficacy beliefs, better general health perception, and higher Work Ability Index score are predictors of (earlier) RTW.

The evidence on gender and educational level being predictors for RTW was inconsistent, and there is evidence from two studies that occupational category is not a predictor for RTW. For many factors the evidence is insufficient because it was identified in only one study, e.g. decision latitude, variety in work, and job demands. Salkever et al. were the only authors who studied benefit plan features [66], and they found that employee mental health benefits and the availability of mental health treatment resources may influence RTW.

#### Recurrent Sickness Absence

In total, 24 factors for recurrent SA in CMD were investigated in 11 included articles [62, 64, 75, 86–93]. Table 4 provides an overview of all 24 factors, categorized in accordance with the framework of the ICF. In the ICF-domains ‘disease related factors’, ‘body functions and structures’, and ‘activities’ no factors were studied.

There is consistent evidence from two studies that having previous episode(s) of sickness absence and shorter duration of employment (tenure) is a predictor of recurrent SA in people with CMDs. There is consistent evidence from seven studies that gender is not a predictor for recurrent SA. In addition, there is evidence from two or more studies that marital status, cohabiting, and full-time work (vs part-time work) are not predictors of recurrent SA. There is inconsistent evidence for age and co-morbidity being predictors for recurrent SA.

### Sub-analysis of the Relation Between CMD Diagnostic Groups and Work Outcomes

In 15 articles in which more than one CMD diagnostic group has been studied, the relation between diagnosis and work outcome was reported (see Supplemental Table 2). Overall, depression appears to be the strongest predictor for worse work outcomes. However, in the three studies that reported about somatoform disorders (somatization), it was concluded that these affected work outcomes even more than depression [57, 73, 79]. In total, 6 studies on SA and five studies on RTW reported different predictive values across diagnoses, and four studies found no differences in RTW or recurrent SA between the different diagnoses.

## Discussion

In this scoping review we provided an overview of predictive factors for (recurrent) SA and RTW among workers with CMDs. Our results indicate that a variety of personal-, work-, and illness related determinants for SA and RTW have been identified so far by research.

### Sickness Absence

In the earlier literature on work outcomes of people with a CMD, the focus of study was mainly on the prognostic value of the condition itself. Since we know that people with a CMD have higher odds to have problems with (sustainable) work participation, but the condition in itself provides an inadequate explanation, the research focus has become more on personal-, and environmental (e.g. work) related factors. The most relevant determinants for SA identified in our review, in terms of association and modifiability, are symptom severity, job demands and control, job strain, organizational (in)justice, sleeping problems, smoking behavior, and perceived general health. Individuals with a CMD with earlier episodes and a past history of absenteeism, who encounter high job demands, low job control, low support at work, sleeping problems, and low perceived health, are at high risk of SA.

There was consistent evidence that earlier episodes of CMD and high symptom severity are predictors for SA. From literature it is known that serious mental disorders are substantially underdiagnosed and undertreated among disability claimants [79, 94, 95], which is associated with inadequate availability and accessibility of care. Early recognition and diagnose of CMDs is very important, especially because interventions might prevent impairment of conditions and work disability. In the end, staying at work might be a powerful determinant for (mental) health of workers with a CMD [96]. However, symptom reduction due to psychosocial interventions is important, but is not a guarantee for reduction of sick-leave [97].

Although it is clear that having a CMD is related with SA, the causality of this relation is less obvious. Sanderson et al. reported that having a CMD was a consequence (and not a risk factor) of SA, limitations in work activities or unfavorable work environment [23]. In other studies, many workers believed that the most important causes of their CMDs were work related, and they reported factors such as work stress, leadership, reduced work participation, job dissatisfaction, work conflict, social work environment, job insecurity and change, workplace bullying, disrupted communication with supervisor, and physical strain [98, 99]. Therefore, preventive interventions for SA “should aim at decreasing psychosocial risk factors for the onset of CMDs at the workplace” [100].

There is consistent evidence that higher perceived job demands combined with low job control is related with SA of workers with CMD [31, 33, 34, 37, 43, 51, 55, 63]. A job with high decision latitude can largely neutralize the risk of high job demands. Therefore, interventions to prevent SA of workers with a CMD should involve the workplace [100]. Improving the work environment might not only prevent SA, it even may prevent the development of a CMD [98, 99].

Earlier episodes of CMD and having a past history of CMD-related SA is a predictor for future SA [24, 28, 38, 60], and therefore in the supervision of absent employees with CMDs more attention should be paid to previous episodes of mental illness. These workers at risk for future SA might be supported to stay at work, although account should be taken of stigmatization of workers.

Poor support or lack of support from the superior (positive feedback and appreciation of achievements) was observed as determinant of SA for workers with a CMD, it doubled the risk of absence for both genders [36]. The authors conclude that improving working conditions, such as social support, “may be an important step toward reducing the burden of SA due to mental conditions”.

### Return to Work

The key determinants for RTW in workers with a CMD currently reported in the literature are symptom severity, duration of previous absenteeism, age, general health perception, bullying, social support from coworkers and supervisor, and positive expectations concerning sick-leave duration or RTW. For most environmental work related factors insufficient evidence was observed (Table 3).

Support from supervisor was variably associated with better work outcomes. Nieuwenhuijsen et al. concluded that supervisors should communicate more frequently with sick-listed employees with CMDs, and hold follow-up meetings more often, as this is associated with a faster RTW in those employees [68]. They advise supervisors to keep in touch with employees who are sick listed at least once every 2 weeks. However, promoting RTW by the supervisor had no effect, and consulting with professionals even had a negative effect on RTW. The explanation of the authors was that “supervisors may consult other professionals sooner if they foresee problems in the RTW-process” [68]. Patients with more social support from coworker or supervisor had a shorter time to RTW [80]. Other studies report no associations between supervisor support and SA [34, 55]. In a recent Swedish study, worse perceived interactional justice with the supervisor was associated with early RTW [82].

We found consistent evidence that the expectations concerning sick-leave duration or RTW are predictive for time to RTW, and may have a significant impact on the outcomes of interventions for RTW. Knowledge of workers’ expectations in the early phase of SA may contribute to shortening the time to RTW, and questioning workers about their expectations can serve as screening the risk of long-time SA [78]. Although expectations about sick-leave duration and RTW have predictive value, an explanation of these expectations should be examined in consultations with the individual employee. Workers’ expectations can be considered as a ‘canary in the coal mine’, and should give rise to a more detailed analysis of both individual- and work-related factors. Workers’ expectations are presumably based on the social context, the available social support both at home and at work, opportunities to realize work accommodations or to return to work gradually, and on the severity of illness. Nieuwenhuijsen et al. reported fatigue, suffering from depression, and workpace and workload as determinants for RTW perceptions [81]. Løvvik et al. reported a strong relationship between illness perceptions and RTW-expectations among people with CMD [101]. Addressing RTW-expectations in occupational healthcare services or vocational rehabilitation might be beneficial in early stages or even prior to a sick-leave episode [102]. Expectations for RTW [103] and self-efficacy [104] can be measured with a questionnaire, although the former needs further validation in a CMD population.

### Recurrent Sickness Absence

About 19–37% of employees with SA due to CMDs at baseline had recurrent episodes after RTW during two year follow-up [86, 92]. It is recommended to follow workers who just returned to work for a longer period and not take their return for granted, because many workers with a CMD have recurrences of SA. The oversight of determinants for recurrent SA does not provide much consistent evidence in favor of certain prognostic factors. This is mainly caused by the fact that most factors were studied only once. The number of previous episode(s) of SA [88, 91] and a shorter tenure [87, 88] were consistently related to recurrent SA. Interventions to prevent recurrence of SA in people with CMDs in order to sustain employees at work, may aim at detection of workers with previous episode(s) of SA and workers with a shorter employment relationship. Furthermore, it sounds reasonable that the predictors for SA may also apply for recurrent SA, and that these could be used too. In one study, it was observed that conflict with supervisor was a risk factor [90]. Obviously, not only the absence of social support from supervisor, but also the presence of negative relationships may affect SA.

### Strengths and Limitations

This scoping review provides a clear overview of the existing empirical evidence about the prognostic factors of SA and RTW among workers with a CMD. A total of 71 articles were identified, which is a far greater range than previously known. The classification of these factors in ICF-domains across work outcomes facilitates retrieval of information and comparison with other research. The data was collected in a systematic manner and the probability of missing important literature is quite low. A strength of this scoping review was that we differentiated between three chronologically occurring work outcomes (SA, RTW, and recurrent SA), and that we presented all applied outcomes.

One general limitation of a scoping review is that no thorough quality assessment of retrieved studies is performed. In order to overcome this limitation, we only presented results established through multivariate analyses, which controlled for possible confounders [105], although the kind of treatment(s) that participants followed was controlled for in only a few of the included studies.

The time to follow-up in the vast majority of studies was sufficient (1 or more years). Although a few studies had a cross-sectional design, in these cases a retrospective data collection was performed on previous treatment, SA, or potential confounders. Another limitation of this scoping review was that CMDs were studied as one group where no distinction was made between different diagnostic groups, such as anxiety disorders and depressive disorders, which makes interpretation less specific. Moreover, in a few studies the exact amount of people with a CMD was unclear. Frequently, more articles were published based on the same cohort study. In these cases, it was not always clear to what extend the research data of these articles overlapped, which occasionally might have led to double reporting.

The majority of studies identified in the review were performed in the Netherlands or the Scandinavian countries. In Denmark, the first period of disability is paid by the local government, the municipalities. In the Netherlands and in Sweden, the employer bears responsibility for sustainable work participation and RTW of employees. In case of continued sickness or disability, Dutch workers get 100% pre-injury earnings compensated by the employer during the first year. This could have the effect that workers are not motivated to get back to work quickly. However, because the employer has incentives and legal obligations to support the absent worker, the possibilities to adapt the work to the needs of workers are utilized when necessary. Thus, in the Netherlands, Denmark and Sweden the employer or the government has an interest in preventing SA and promoting RTW, and initiates interventions to succeed. In this context, it is understandable that a boom of research on SA and RTW was initiated in these countries. However, the question is to what extent these results can be generalized to other countries. A compensation policy that provides for economic support in case of sickness or disability does not exist in all countries. Thereby, the propensity to take sick-leave or to return to work will differ across jurisdictions, even across Australian state and territory workers’ compensation systems [106]. In the USA, social security disability insurance taxes may discourage individual firms from investing in RTW. Likewise, the jurisdictions for CMDs as accepted cause of SA differ across countries. Therefore, it is not easy to draw general conclusions about predictive factors for SA or RTW, because a promoting factor in one jurisdiction might be a limiting factor in another.

### Gaps in the Current Knowledge

Factors from the activities domain of the ICF framework are under investigated. Probably, researchers think that activity level is unimportant or not relevant in people with a CMD because they have no activity limitations. On the other hand, inactivity is an important symptom in CMDs and is not conducive to recovery [107]. From this point of view, the relation of (in)activity should be analyzed more in future research. In our review we identified two articles which studied the relation between physical activity and SA, of which one concluded that physical activity was related [40] and the other found no association [63]. There is currently not enough evidence to draw conclusions about activities-related factors as determinant for SA or RTW.

Salkever et al. are the only authors who focused their study on benefit plan features, such as availability of mental health benefits and services, employers’ disability management practices, and long-term disability policy provisions provided by the employer [66]. It was concluded that integration of disability management with related services, and providing job accommodations was related to a higher probability of RTW. Provision of more generous benefits in terms of lower deductible, shorter preexisting condition exclusion period, and not having a carve out encouraged earlier RTW. Employees having a broader criterion for continuing disability had a lower probability of RTW. Because the effect of benefit plan features was only studied by Salkever et al., the evidence was classified as insufficient. Notwithstanding, the results are interesting. It was observed that benefit plan features may play a role in the RTW trajectories of workers with CMDs. This study demonstrates that the benefit plan features, which may differ across companies, individuals, and even countries, could possibly affect SA and RTW. To what extent these results are also valid in other benefit systems in countries other than the USA, where jurisdictions and legislation are different, is still unclear.

Organizational justice was observed as determinant for SA in people with a CMD [50, 54]. In a largely representative sample of employees in the Netherlands, it was found that both distributive and procedural justice contributed to lower depressive symptoms, and distributive justice contributed to lower SA [108]. Perceived injustice in general might be an important determinant, which is already studied among people with musculoskeletal problems [109]. Emerging evidence suggests that perceived injustice might be a relevant factor for many people with chronic non-specific back pain and is considered as a determinant for work disability [109, 110]. The feelings of injustice may be directed against the employer, the insurer, co-workers, healthcare workers, occupational physician, or the person who performed a functional capacity evaluation. Because perceived injustice is likely related to depressive feelings [108, 111], it could be addressed in future research and practice of CMDs.

In a systematic review exploring illness perception in mental health utilizing the self-regulation model, it was concluded that the dimensions of the self-regulation model were largely supported, and applicable to mental illness [112]. We did not find sufficient evidence in our review about illness perceptions as predictor for work outcomes among people with CMDs. Illness perceptions are derived from the self-regulatory model of health behavior [113], which provides a framework for understanding the processes by which an individual’s own implicit, common-sense beliefs about illness are associated with behavioral responses employed to manage outcomes. Five dimensions of illness perceptions are distinguished: identity (the label of the illness and the symptoms the patient views as being part of the disease); cause (personal ideas about etiology); time-line (how long the patient believes the illness will last); consequences (expected effects and outcome of the illness); and cure/control (how one recovers from, or controls, the illness) [113]. Løvvik et al. found that illness perceptions predicted benefit recipiency in people with CMDs in the unadjusted model, but not in the fully adjusted model [114]. Results from a recent systematic review suggest that illness perceptions may play an important role in mediating between illness and work outcomes [115]. Although expectations about recovery as earlier described in our review is part of illness perceptions, there are more interesting aspects of illness perceptions mentioned above which have not been studied yet in CMDs.

To what extent are the prognostic factors in the present study congruent to the opportunities and obstacles mentioned to be important for work outcomes by the workers with CMD themselves? In a meta-analysis of qualitative research on RTW among employees with CMDs, a number of obstacles and facilitators were identified [116]. Perfectionist character made it difficult for employees to slow down their work pace and to accept reduced work capacity. The possibility to gradually RTW (increase working hours, responsibilities, and workload), the realization of work accommodations, and social support from both supervisors and co-workers were reported as important facilitators by employees. Attitudes of employers and co-workers towards sick-leave and development of CMD affected the motivation to return to work significantly. Perceived injustice may occur when understanding of CMD symptoms is lacking, and when work accommodations are not acknowledged and respected [116]. The right timing for RTW, in other words the readiness for RTW, was indicated as an important factor. In a qualitative study, the RTW process of workers on sick leave due to CMD was studied [117]. The main perceived barriers experienced by the workers were the inability to set limits, decreased capacity, recognition of exhaustion, lack of support, and to control cognitions and behavior such as perfectionism. Indicated solutions were learning a new way of dealing with work demands, and treating mental or physical symptoms. Furthermore, an intention-behavior gap was observed between the solutions and intentions to full RTW. According to the authors, having a positive attitude and increasing self-confidence by extending the workload carefully towards a full RTW are prerequisites for the intent to proceed.

In a Delphi study, group consensus was sought among scientists and physicians with expertise in assessing work disability on factors predicting recurrent SA due to depression [118]. Workers at risk of recurrent SA due to depression may be identified by stressful life and work events, number and duration of earlier depressive episodes, psychological work demands, decision latitude, and commitment to work.

### Recommendations for Future Research

In this scoping review we provided an oversight of prognostic factors for (recurrent) SA and RTW among people with a CMD. Further research is clearly required; there is a need for a systematic review or meta-analysis, in which the strength of prognostic values is investigated. The following factors are regarded as important by workers with CMDs and should be considered as subject for future primary research, because original primary studies are lacking: perfectionism, illness perceptions, acceptance of the illness and decreased work capacity, the possibility for gradual RTW and work accommodations, and perceived injustice. There is a need to develop more interventions to prevent SA and to improve RTW for workers with a CMD, and also to carry on studies that investigate its effectiveness. When future interventions are designed based on the known prognostic factors for SA and RTW, their effectiveness can potentially be improved.

More research on predictors for RTW in people with CMDs is needed in the ICF-domains ‘body functions and structures’, ‘activities’, and ‘environmental factors’. Concerning SA, RTW and recurrent SA, more research is needed on ‘environmental (work related) factors’, because the evidence in this domain was mostly insufficient as it came from only one study. Lastly, more research is needed on recurrent SA, because prognostic factors in the ICF-domains ‘disease related factors’, ‘body functions and structures’, and ‘activities’ were lacking.

## Conclusions

The amount of research on determinants for SA and RTW in workers with CMD has increased dramatically in recent years. It is noticed that the majority of studies has been carried out in the Scandinavian countries and the Netherlands. A variety of personal-, work-, and illness-related determinants have been observed across the ICF domains. Although illness related factors are playing an important role in SA and RTW of workers with CMDs, health interventions alone are insufficient to prevent SA and to improve RTW. Symptom reduction due to psychosocial interventions does not automatically result in a reduction of sick-leave. Work-related interventions are essential and should always be part of a prevention or reintegration program. In care for people with CMDs, the management of expectations should be taken very seriously, because expectations often reveal issues in environmental and personal domains that bother the individual worker. Future interventions for improvement of work outcomes should be built with a variety of prognostic factors from different domains.

There is a lack of studies in the ICF domains ‘activities’ and ‘environmental factors’. In addition, there are some research gaps identified in this scoping review that need further attention in primary and secondary studies.

## Electronic supplementary material

Below is the link to the electronic supplementary material.

Supplementary material 1 (DOCX 127 KB)

Supplementary material 2 (DOCX 18 KB)
