# Supplementary material for: Determinants of Sickness Absence and Return to Work Among Employees with Common Mental Disorders: A Scoping Review
Source: J Occup Rehabil. 2017 Oct 4;28(3):393–417. doi: 10.1007/s10926-017-9730-1 (PMC6096498; doi:10.1007/s10926-017-9730-1)
Supplement: Supplementary file 1 — Supplementary material 1 (DOCX 127 KB) [file 10926_2017_9730_MOESM1_ESM.docx]

**Supplemental Table 1a - 1c: Included articles respectively on sickness absence, return to work and recurrent sickness absence.**

**Table 1a: Included articles on prognostic factors for sickness absence in people with a Common Mental Disorder**

| **No.** | **Author / Year /**  **Country** | **Type of study /**  **Follow-up / Aim** | **Study**  **population** | **Prognostic factors studied** | **Outcome** | **Association estimates (95% CI)** |
| --- | --- | --- | --- | --- | --- | --- |
| 1 | Souêtre et al. [24]  1997  France | Cross-sectional, with retrospective data collection (1 year) on previous treatment, sickness absence, or potential confounders.  This study examines the predicting factors for absenteeism in depressed patients. | 345 patients diagnosed with major depressive disorder (MDD) | Treatment with Fluoxetine,  Tricyclics | Absenteeism measured as:  current absenteeism versus no absenteeism (absenteeism defined as at least one day absent). | For all patients, the strongest  predictors of absenteeism from work were:  Symptom severity OR= 44.4 (7.9–250)  Past history of depression OR=6.85 (2.6–18.4)  Past history of absenteeism  OR= 6.51 (2.0–204) |
| 2 | Laitinen-Krispijn & Bijl [25]  2000  The Netherlands | Cohort study.  Follow-up 1 year.  To determine to  what extent different forms of mental disorders are related to an increased likelihood of sickness absence and whether this increase depends on the employee's sex. | Men (n = 2,064) and Women (n = 1,631) individuals with mental disorders (depression, anxiety)  Netherlands Mental Health Survey  and Incidence Study (NEMESIS) | Gender  Major depressive disorder  Dysthymia  Any mood disorder  Panic disorder  Social phobia  Simple phobia  Any anxiety disorder  Alcohol abuse  Alcohol dependence  Drug abuse/dependence  Any substance use disorder  Any DSM-III-R disorder | Subsequent sickness absence (at least one sickness absence spell during the last 12 months; the duration of this spells were unknown). | Mood disorders  Major depressive disorder  Men: OR=1.87 (1.23;2.82)  Women: OR=1.29 (0.80;2.08)  Dysthymia  Men: OR=2.97 (1.19;7.41)  Women: OR=1.13 (0.61;2.10)  Any mood disorder  Men: OR=1.72 (1.20;2.48)  Women: OR=1.20 (0.81;1.79)  Anxiety disorders  Panic disorder  Men: OR=1.25 (0.49;3.19)  Women: OR=1.22 (0.61;2.41)  Social phobia  Men: OR=1.05 (0.65;1.69)  Women: OR=0.64 (0.37;1.09)  Simple phobia  Men: OR=2.01 (1.22;3.32)  Women: OR=1.10 (0.79;1.53)  Any anxiety disorder  Men: OR=1.37 (1.00;1.88)  Women: OR=0.94 (0.70;1.27) |
| 3 | Druss et al. [26]  2000  United States | Cross-sectional, with retrospective data collection (1 year) on previous treatment, sickness absence, or potential confounders.  This study compares  the health and disability costs of depressive illness with those of four other chronic conditions among employees of a large U.S. corporation. | 9,398 individuals who filed at least one  claim for depressive illness | **Depressive disorder:**  Diagnosis of major depression, dysthymia, or depressive disorder  **Comorbidities**  Diabetes  Heart Disease  Hypertension  Back Problems  **Sociodemographic characteristics**  Age  Race  Sex  Annual income  Tenure of employment Education level  The state in which they were employed | Number of annual sick days. | **Model 2:**  Diabetes, heart disease, hypertension,  or back problems only (N=1,956)  mean=6.64 t=12.50 p-value=<0.001  Depressive disorder only (N=312) mean=8.79 t=3.19 p-value=0.01  Both (N=100)  mean=13.48 t=5.44 p-value=<0.001 |
| 4 | Lerner et al. [27]  2004  United States | Cross-sectional, with retrospective data collection (2 weeks) on previous treatment, sickness absence, or potential confounders.  The study determined whether depression’s negative productivity impact varied with the type of work employees performed. | 246 employees with depression (dysthymia = 64; MDD = 89 and DD = 93) and 143 healthy controls | Occupation type  0*NET occupational requirements  Depression severity  PHQ-9 depression symptoms  PCS 12 physical health  Age  Gender  Education | Number of workdays missed in the past 2 weeks. | **General model:**  **Occupation**  Sales, service and support occupations  B=0.2; S.E.=0.2 (−0.2; 0.6)  **Production, construction, repairs and trans, occupations**  B=−0.2; S.E.=0.4 (−1.1; 0.6)  **Depression severity**  B=2.2; S.E.=0.5 (1.4; 3.2)  **Physical health** (PCS 12)  B=−3.1; S.E.=0.8 (−4.7; −1.5)  **Age**  B=0.5; S.E.=1.0 (−1.4; 2.4)  **Gender: male**  B=0.1; S.E.=0.3 (−0.4; 0.6)  **Specific Model:**  **Occupational requirements**  Judgement/Communication skills (0–1)  B=1.3; S.E.=0.9 (−0.5; 3.1)  External customers (0–1)  B=0.6; S.E.=0.5 (−0.3; 1.5)  **PHQ-9 depression symptoms**  Concentrate/Fidget (0–1)  B=0.7; S.E.=0.5 (−0.3; 1.7)  Tired/Sleep Problems (0–1)  B=1.0; S.E.=0.4 (0.2; 1.9)  PCS-12 physical health(0–1)  B=−3.1; S.E.=-0.9 (−4.9; −1.3)  **Age**  B=−0.1; S.E.=1.1 (−2.1; 2.0)  **Male**  B=−0.1; S.E.=0.3 (−0.6; 0.4)  **Education**  B=−0.2; S.E.=0.1 (−0.3; −0.1) |
| 5 | Rytsälä et al. [28]  2005  Finland | Cross-sectional.  The aim of this study was to investigate predictors of level of psychosocial disability and current work disability in a large sample of patients with MDD. | 269 psychiatric inpatients and outpatients  with MDD. | Gender  Age  Hamilton-Depression Scale  Number of episodes of depression | Sick Leave (being at work or on sick leave) | Female sex  OR=3.9502 (1.65;9.46)  Age  OR=1.0534 (1.01;1.09)  Severity of depression (Ham-D)  OR=1.0796 (1.01;1.16)  Number of previous episodes of depression  OR=1.5821 (1.03;2.43) |
| 6 | Buist-Bouwman et al. [29]  2005  The Netherlands | Cohort study  Follow-up 1 year  To examine the association between physical and mental  disorders and the separate and joint eﬀect of physical and mental disorders on work-loss. | 7076 persons  The following DSM-III-R diagnoses were included in this study: anxiety disorder, mood disorder and substance use disorder.  Netherlands Mental  Health Survey and Incidence Study (NEMESIS) | **Mental Disorders**  Anxiety disorders  Mental disorders  Mood disorders  Substance abuse disorders  **Physical disorders**  Sinus infection  Chronic back trouble Rheumatism  Hypertension  Asthma  Migraine  Digestive system  Accidental Injury  Age  Gender  Education | Number of workdays lost during the last 12 months. | **Joint Effects:** Associations involving  comorbid conditions  Physical–mental comorbidities (CO)  Sinus infection OR=17.8; S.E= (2.5)***  Chronic back trouble OR=44.3; S.E= (2.6)***  Rheumatism OR=32.7 ;S.E= (2.8)***  Hypertension OR=28.3; S.E= (2.9)***  Asthma OR=18.6; S.E= (3.2)***  Migraine OR=27.6; S.E= (3.2)***  Digestive system OR=33.3; S.E= (3.7)***  Accidental Injury OR=27.5; S.E= (4.3)*** |
| 7 | Moriana and Herruzo [30]  2006  Spain | Cross-sectional  This study analyses the relationships of certain socio-demographic, organizational, and personality variables with psychiatric sick leave taken by Spanish secondary school teachers. | 200 teachers, 100 with a psychiatric sick-leave (54.5% anxiety and 44.8% depression) and 100 controls. | Gender  Marital status  Degree (humanities or sciences)  Job status  Cigarette smoking  Alcohol consumption  Anxiety  Somatic symptoms  Depression  Total Experimental Index  Competitiveness  Work overload  Impatience  Hostility  Total JASE-H  Job satisfaction  Emotional exhaustion  Depersonalization  Personal accomplishment  Total MBI-E | Presence of psychiatric sick-leave (versus no presence of sick-leave) | **Coefficient / S.E.**  Competitiveness  ß=0.0645 / SE=0.02 ; p=0.009  Hostility  ß=0.0433 / SE=0.03; p=0.155  Job satisfaction  ß=0.0708 / SE=0.01; p=0.001  Emotional exhaustion  ß=0.0597 / SE=0.01; p=0.001 |
| 8 | Melchior et al. [31]  2007  France | Cohort study  Follow-up 9 years  We studied the association between multiple work and family demands and sickness absence due to non-psychotic  psychiatric disorders | 8,869 men and 2,671 women from the GAZEL study.  From 1997 to 2003, diagnoses were coded following the 10th version of the ICD: depressive episode (F32), recurrent depressive disorder (F33), persistent mood  disorder (F34), phobic anxiety disorders (F40), other anxiety disorders  (F40–F41), other neurotic stress-related or somatoform  disorders (F42–F44), organic, including symptomatic mental disorders  (F00–F09), disorders due to the use of alcohol (ICD-10: F10)  or other drugs (ICD-10: F11–F19), other mental disorders. | **Work stress factors**  **Number of dependents**  (Dependents are all individuals who do not have an income and are  financially supported by the employee (typically children or the  spouse).  **Covariates**  Age  Occupational grade  Marital status  Personal social support  Life events  Alcohol consumption  Body mass  Depressive symptoms at baseline | Psychiatric SA  (days of sickness absence) | **(Fully adjusted models for men and women)**  ***All non-psychotic psychiatric diagnoses group***  **Work stress factors + n dependents**  **0 (no dependent)**  Men: RR=1.85 (0.74;4.64)  Women: RR=2.95 (1.61;5.42)  **1 (ref)**  **2 dependents**  Men: RR=1.03 (0.63;1.67)  Women: RR=1.52 (0.98;2.37)  **3 (2 or more work stress factors + 4 or more dependents)**  Men: RR=1.82 (0.86;3.87)  Women: RR=5.04 (2.84;8.90)  ***Depression group***  **Work stress factors + n dependents index**  **0 (no dependent)**  Men: RR=0.53 (0.07;4.28)  Women: RR=1.81 (0.80;4.11)  **1 (ref)**  **2 dependents**  Men: RR=1.12 (0.59;2.10)  Women: RR=1.48 (0.87;2.51)  **3 (2 or more work stress factors + 4 or more dependents)**  Men: RR=3.55 (1.62;7.77)  Women: RR=6.58 (3.46;12.50) |
| 9 | Vaez et al. [32]  2007  Sweden | Cohort study  Prospective and retrospective data from 1996–2002  The aim of this study was to investigate sickness absence and disability pension in a cohort of employees who initially were on long-term sick leave due to psychiatric disorders with regard to gender, age, socioeconomic status, and previous sickness absence. | 4,891 employees, who had a new sick-leave spell >90 days with a psychiatric disorder.  The main diagnoses were depression, anxiety, and  stress disorders (F32, F41, and F43), burnout (Z73) and exhaustion (T73). | **Gender**  **Age (in years)**  20–34  35–44  45–54  55–61  **Socioeconomic status**  High/intermediate non  manual employees  Assistant non-manual  employees  Skilled manual workers | Number of sick-leave days  <17 days  17–90 days  91–365 days | **(Adjusted model for all covariates for men and women)**  ***Gender***  Women (ref)  Men  **<17 days:** OR=1.12 (0.96;1.31)  **17–90 days:** OR=0.99 (0.77;1.29)  **91–365 days:** OR=0.88 (0.74;1.03)  **DP**: OR=1.03 (0.87;1.22)  ***Age (years)***  **20-34 (ref)**  **35–44**  <17 days: OR=0.90 (0.75;1.08)  17–90 days: OR=0.68 (0.52;0.90)  91–365 days: OR=1.02 (0.84;1.23)  DP: OR=1.47 (1.15;1.88)  **45–54**  <17 days: OR=0.65 (0.54;0.78)  17–90 days: OR=0.65 (0.49;0.85)  91–365 days: OR=0.90 (0.74;1.08)  DP: OR=2.72 (2.15;3.43)  **55–61**  <17 days: OR=0.42 (0.34;0.52)  17–90 days: OR=0.48 (0.34;0.68)  91–365 days: OR=0.61 (0.48;0.76)  DP: OR=6.30 (4.80;8.09)  ***Socioeconomic status***  **High/intermediate non-manual employees** (ref)  **Assistant non-manual employees**  <17 days: OR=0.63 (0.47;0.84)  17–90 days: OR=1.17 (0.66;2.07)  91–365 days: OR=1.17 (0.85;1.62)  **Skilled manual workers**  <17 days: OR=0.51 (0.38;0.69)  17–90 days: OR=1.50 (0.85;2.66)  91–365 days: OR=1.02 (0.74;1.41)  ***Unskilled manual workers***  <17 days: OR= 0.38 (0.28;0.52)  17–90 days: OR=1.37 (0.77;2.45)  91–365 days: OR=0.93 (0.67;1.29) |
| 10 | Virtanen et al. [33]  2007  Finland | Cohort study  Follow-up 2 years  This prospective  study was assessed using the General Health Questionnaire (GHQ-12), which identified  psychologic distress as a predictor of sickness absence and the effect of work-unit measures of job strain on sickness absence among all cases. | 7,986 public workers with psychological distress (mood and anxiety disorders) | **Gender**  **Job demands (component of strain)**  Low  Intermediate  High  **Job control (component of strain)**  High  Intermediate  Low  **Job strain**  Low strain  Passive  Active  High strain  **Socioeconomic position** | Long term SA  (more than 7 days clinically certified SA) | **Job demands (component of strain)**  Women  Low: RR=1.00  Intermediate: RR=1.06 (0.97;1.16)  High: RR=1.17 (1.07;1.27)  Men  Low: RR=1.00  Intermediate: RR=1.03 (0.85;1.26)  High: RR=1.03 (0.81;1.30)  **Job control (component of strain)**  High  Women: RR=1.00  Men: RR=1.00  Intermediate  Women: RR=1.11 (1.02;1.22)  Men: RR=1.26 (0.99;1.60)  Low  Women: RR=1.14 (1.04;1.25)  Men: RR=1.46 (1.17;1.81)  **Job strain**  Low strain  Women: RR=1.00  Men: RR=1.00  Passive  Women: RR=1.05 (0.94;1.16)  Men: RR=1.09 (0.87;1.38)  Active  Women: RR=1.09 (0.99;1.21)  Men: RR=0.82 (0.63;1.08)  High strain  Women: RR=1.17 (1.06;1.29)  Men: RR=1.41 (1.08;1.83)  **Low socioeconomic position**  **Job strain:**  Low strain  Women: RR=1.00  Men: RR=1.00  Passive  Women: RR=0.96 (0.84;1.09)  Men: RR=1.14 (0.85;1.53)  Active  Women: RR=1.10 (0.95;1.28)  Men: RR=0.68 (0.44;1.03)  High strain  Women: RR=1.06 (0.93;1.21)  Men: RR=1.31 (0.93;1.85)  **High socioeconomic position**  **Job strain:**  Low strain  Women: RR=1.00  Men: RR=1.00  Passive  Women: 1.36 (1.14;1.62)  Men: 1.09 (0.75;1.59)  Active  Women: 1.16 (1.01;1.33)  Men: 0.91 (0.63;1.32)  High strain  Women: 1.54 (1.33;1.77)  Men: 1.58 (1.05;2.37) |
| 11 | Sanderson et al. [23]  2008  Australia | Cohort study  Follow-up 6 months  This prospective study used the framework of ICF components to investigate the magnitude and direction of association between body functions (depression/anxiety symptoms), activity (limitations in work activities), participation  (sickness absence), and environment (psychosocial aspects) in the workplace setting. | 204 employees with any physical or mental disorder | **International Classification of Functioning, Disability and Health - ICF components:**  Depression/anxiety  Limitations in work activities  Work environment  **Covariates:**  Gender  Age  Education | Sickness absence: number of days absent from work in the past 4 weeks due to health. | **(Model adjusted for baseline + covariates)**  **Depression/anxiety as risk factor for SA:** RR= 1.0 (0.7 – 1.4) p value=0.97  **Limitations in work activities as risk factor for SA:**  RR= 1.1 (0.8 – 1.5) p value= 0.52    **Work environment as risk factor for SA:** RR= 1.1 (0.9 – 1.5) p value= 0.35 |
| 12 | Clumeck et al. [34]  2009  Belgium | Cohort study  Data collection: 1994 and 1998  Belstress I study on the relationship between perceived job stress and health problems.  In this study, the impact of adverse psychosocial working conditions is analyzed on the risk for long-term sick leave due to depression. | Employees selected from 11 large companies (n=9,396) | **Karasek stress dimensions**  Job control  Psychological demand  Social support  **Covariates:**  Age  Gender  Living situation  International Standard Classification of Occupations (ISCO)  Center of Epidemiological Studies-Depression (CESD) scale - CESD at baseline | Incidence of long-term sick leave for depression (sick leave ≥ 28 days) | ***Men***  **Strain**  Low strain: OR=1  Active: OR=1.72 / (0.72;4.12)  Passive: OR=2.67 / (1.15;6.19)  High strain: OR=3.23 / (1.40;7.43)  **Iso-strain**  No H strain/H support: OR=1  No H strain/L support: OR=1.39 / (0.81;2.40)  H strain/H support: OR=2.08 / (1.04;4.16)  H strain/L support: OR=1.94 / (1.06;3.54)  ***Women***  **Strain**  Low strain: OR=1  Active: OR=0.91 / (0.38;2.22)  Passive: OR=1.48 / (0.65;3.38)  High strain: OR=1.77 (0.79;3.95)  **Iso-strain**  No H strain/H support: OR=1  No H strain/L support: OR=0.83 / (0.45;1.54)  H strain/H support: OR=1.27 / (0.67;2.42)  H strain/L support: OR=1.44 / (0.80;2.58)  ***Men***  **Psychological demands**  Low: OR=1  Medium: OR=1.73 / (1.01;2.96)  High: OR=1.23 / (0.66;2.27)  **Job control**  High: OR=1  Medium: OR=1.77 / (0.92;3.44)  Low: OR=2.43 / (1.27;4.66)  **Social support**  High: OR=1  Medium: OR=0.56 / (0.29;1.07)  Low: OR=0.86 / (0.49;1.50)  ***Women***  **Psychological demands**  Low: OR=1  Medium: OR=1.03 / (0.60;1.77)  High: OR=1.06 / (0.60;1.89)  **Job control**  High: OR=1  Medium: OR=1.02 (0.46;2.26)  Low: OR=2.21 (1.05;4.68)  **Social support**  High: OR=1  Medium: OR=1.18 (0.64;2.18)  Low: OR=0.91 (0.49;1.68) |
| 13 | Lexis et al. [35]  2009  The Netherlands | Cohort study  Follow-up 10 months  This study aims to evaluate the relationship between depressive complaints and sickness absence in the working population. | 3,339 screened with the Hospital Anxiety and Depression (HAD-D) Scale | **Depressive complaints**  Normal range  Mild complaints  Moderate and severe complaints  **Covariates:**  Age  Gender  Educational level  Living alone  Shift work  Long-term illness  Smoking | SA (yes versus no)  SA duration (total number of days absent from work over the 10 months of follow-up) | **Outcome: SA (yes versus no)**  ***HAD-D score***  **Men**: OR=1.15 (1.11-1.19)  **Women**: 1.16 (1.11-1.21)  ***Severity***  **Men**  Mild complaints: OR=2.21 (1.56;3.15)  Moderate-severe complaints: OR=4.00 (2.76;5.81)  **Women**  Mild complaints: OR=1.73 (1.00;2.98)  Moderate-severe complaints: OR=5.33 (3.20;8.88)  ***HR / CI 95%***  ***Outcome: Shorter Time to Onset of First Sickness Absence Spell***  **Men**  Mild complaints: HR=1.20 (0.98;1.46)  Moderate-severe complaints: HR=1.57 (1.22;2.02)  **Women**  Mild complaints: HR=1.36 (0.93;1.99)  Moderate-severe complaints: HR=1.56 (0.99;2.45)  ***Coefficient / CI 95%***  ***Outcome: SA duration***  **Depressive complaints**  Men: ß=0.0735 (0.0549;0.0921)  Women: ß=0.0730 (0.0366;0.1095) |
| 14 | Foss et al. [36]  2010  Norway | Cohort study.  Follow-up 5 years (The Oslo Health Study).  To identify individual and work-related predictors of long-term (8 weeks) sickness absence with psychiatric diagnoses (LSP). | 8,333 subjects  During follow-up, 344 (7.8%) women and 153 men (3.9%)  experienced at least one LSP (depressive disorder (n=241), anxiety disorder (n=18), affective disorder (n=7), neurasthenia (n=13), psychosis (n=6) | Age (yr)  Education  Support from superior  General health  Mental distress  Smoke cigarettes  Alcohol problem | **H**aving at least one spell of long-term (>8 weeks) sickness absence due to psychiatric sickness | **Men**  **Age (yr)**  40: HR=1.7 (1.1;2.5)  45: HR=1.7 (1.1;2.6)  **Education**  1: HR=1.0 (0.5;2.0)  2: HR=0.9 (0.5;1.6)  3: HR=1.0 (0.6;1.6)  4: HR=0.7 (0.4;1.2)  Missing: HR=1.0 (0.4;2.4)  **Support from superior**  Poor: HR=1.6 (1.0;2.4)  Missing: HR=0.5 (0.1;2.5)  **General health**  Good: HR=1.2 (0.8;2.0)  Poor: HR=1.8 (1.0;3.2)  Missing: HR=3.9 (1.3;11.9)  **Mental distress**  Yes: HR=2.7 (1.7;4.3)  Missing: HR=1.7 (0.9;3.3)  **Smoke cigarettes**  Never (ref.)  Yes, before: HR=0.7 (0.4;1.1)  Yes, now: HR=1.3 (0.9;1.9)  Missing: HR=1.1 (0.3;4.8)  **Alcohol problem**  Yes: HR=2.3 (1.4;3.7)  Missing: HR=2.2 (0.6;8.5) |
| 15 | Kivimaki et al. [37]  2010  Finland | Cohort study  Follow-up 12 months  To examine unbiased associations between self-reported job demands and sickness  absence with a psychiatric diagnosis | 2,784 female nurses working in somatic illness wards.  102 nurses had an absence with a psychiatric diagnosis:  64 neurotic stress-related and somatoform  disorders (ICD-10 codes F40–F48), 32 mood disorders  (ICD-10 codes F30–F39), 4 behavioral syndromes (ICD-10 codes F50–F59), and 2 ‘‘other’’ mental or behavioral disorders | Hospital ward overcrowding  score  Job demands score  Job control score  **Covariates:**  Age  Employment contract  Permanent  Temporary  Length of Employment years  Specialty  Internal medicine  Surgery  Pediatrics  Other  Smoking (No/Yes)  Physical inactivity (No/Yes)  Alcohol intake of >210 g/week (No/Yes)  Body mass index | Sickness absence due to a psychiatric diagnosis (yes or no) | **Outcome: any mental or behavioral disorder (Probit model)**  Exposure to overcrowding (range, 1–4)  ß=0.146 (0.065;0.227)  Job demand score (range, 1–5)  ß=0.222 (0.106;0.339) |
| 16 | Van der Werff et al. [38]  2010  The Netherlands | Cross-sectional, with retrospective data collection (6 months) on previous treatment, sickness absence, or potential confounders.  NESDA study  Aim of the present study was to examine the effect of illness characteristics and comorbid mental disorders on various aspects of disability  among persons with a current MDD episode. | 607 participants with a current MDD  -analysis for 332 patients who were working | Gender  Age  **Illness characteristics**  MDD symptom severity  Melancholic features  Atypical features  Manic features  Time depressed last 5 years  Recurrence  Depression age of onset  Dysthymia diagnosis  **Comorbid mental disorders**  Panic disorder  Social phobia  GAD  Agoraphobia  Alcohol dependence | **Work absence**  Work weeks absent from work during the last 6 months (work absence was computed by dividing the number of days absent by the number of days supposed to work; range 0-26) | **Sociodemographics**  Gender: Female vs. Male  Multivariate: ß=−.04  Age: Multivariate: ß=.01  **Illness characteristics**  MDD symptom severity: ß=.30***  Melancholic features: ß=−.06  Atypical features: ß=−.004  Manic features: ß=.01  Time depressed last 5 years: ß=.03  Recurrence: ß=.06  Depression age of onset: ß=.18*  Dysthymia diagnosis: ß=.06  **Comorbid mental disorders**  Panic disorder: ß=.01  Social phobia: ß=−.04  GAD: ß=−.04  Agoraphobia: ß=.07  Alcohol dependence: ß=−.01  * p-value .05 / ** p-value .01 / *** p-value .001 |
| 17 | Catalina-Romero et al. [39]  2011  Spain | Prospective cohort study | 1,292 workers with depression | Gender  Age  Education level  Payment modality  Specialist treatment | SA duration | **Gender**  Men vs. Women  OR=0.69 (0.53;0.89) p-value=0.005  **Age**  25-34 vs. < 25 years: OR=2.71 (1.63;4.49) p-value=0.0001  35-44 vs. < 25 years: OR=3.17 (1.9-5.3) p-value=< 0.0001  45-54 vs. < 25 years: OR=3.49 (2.04-5.98) p-value=< 0.0001  > 55 vs. < 25 years: OR=4.61 (2.45-8.7) p-value=< 0.0001  **Education level**  Primary vs. No studies: OR=0.49 (0.22-1.09) p-value=0.08  Secondary vs. No studies: OR=0.47 (0.21;1.06) p-value=0.07  Medium vs. No studies: OR=0.36 (0.15;0.84) p-value=0.02  University vs. No studies: OR=0.32 (0.13;0.79) p-value=0.01  **Payment modality**  Delegate payment vs. direct payment by employee: OR=0.63 (0.45;0.89) p-value=0.009  Delegate payment by another person vs. direct payment by employee: OR=1.64 (0.99;2.71) p-value=0.05  **Specialist treatment**  Psychiatrist vs. No specialist: OR=1.27 (0.94;1.71) p-value=0.12  Psychologist vs. No specialist: OR=1.46 (0.79;2.73) p-value=0.23  Others vs. No specialist: OR=0.94 (0.05;18.95) p-value=0.97 |
| 18 | Verboom et al. [40]  2011  The Netherlands | Cross-sectional, with retrospective data collection (6 months) on previous treatment, sickness absence, or potential confounders.  NESDA study  This study aims to  examine the effects of personal and environmental characteristics on disability with MDD, over and above illness characteristics. | 573 patients with a current diagnosis of MDD (265 working participants) | **Personal characteristics**  Age  Gender  Years of education  Personality  neuroticism  extraversion  openness  agreeableness  conscientiousness  Chronic diseases  Physical activity:  low  moderate  high  Family history  **Environmental characteristics**  Childhood trauma  Adverse life events  Social network size  Partner support  no partner  low support  moderate support  high support  Friend support:  no friends  low support  moderate support  high support  Household income  Stress at work:  low stress  moderate stress  high stress  **Illness characteristics**  MDD symptom severity  Age of onset of MDD  Agoraphobia  Alcohol dependence | **Work absence:** the number of days absent from work during the last six months divided by the number of workdays the participant was supposed to work in the last six months | Outcome: work absence (model 5 including all significant covariates)  **Personal characteristics**  Age ß=0.054  Gender ß=-0.012  Years of education ß=0 .000  Personality  Neuroticism ß=-.179  Extraversion ß=-.113  Openness ß=-0.056  Agreeableness ß=0.053  Conscientiousness  ß=-0.124  Chronic diseases ß=0 .058  Physical activity  Moderate ß=0.009  High ß=-0.137  Family history ß=-0.042  **Environmental characteristics**  Childhood trauma ß=0.040  Adverse life events ß=0.053  Social network size ß=0.005  Partner support  low support: ß=0.003  moderate support: ß=0.054  high support: ß=-0.001  Friend support  low support: ß=-0.091  moderate support: ß=0.065  high support: ß=-0.028  Household income: ß=-0.083  Stress at work  moderate stress: ß=0.101  high stress: ß=0.155  **Illness characteristics**  MDD symptom severity: ß=0.282  Age of onset of MDD: ß=0.080  Agoraphobia: ß=0.067  Alcohol dependence: ß=0.006 |
| 19 | Stansfeld et al. [41]  2011  Great Britain | Cohort study  Follow-up :  Phase 1: 1985-1988  Phase 2: 1989-1990  Phase 3: 1991-1998  The study aims to:  - examine whether sub-clinical as well as clinical psychiatric morbidity predict long spells of sickness absence for both psychiatric and non-psychiatric illness.  - examine whether recent common mental disorders and those present on two occasions have a stronger association with sickness absence than less recent and single episodes of disorder. | 5,104 civil servants from the longitudinal Whitehall II Study | Common Mental Disorder diagnosis at phases 1 and 2 through the use of the GHQ caseness.  **Covariates**  Age  Employment grade  Work characteristics  Decision latitude  Job demands  Work support  Effort reward imbalance  Marital status  Social support  Confiding/emotional  Practical  Negative aspects  Network outside household  Material problems  Alcohol consumption  Self-reported health  Health problems in last year  Presence of longstanding illness  Presence of physical illness | Spells of sickness absence (1991-1998) | Variable: GHQ caseness  ***Men***  **Psychiatric long spells**  0-2 months: RR=1  3-4: RR=0.99 (0.53 to 1.87)  5+: RR=1.67 (1.13 to 2.46) |
| 20 | Peterson et al. [42]  2011  Sweden | Cohort study.  Follow-up 3.5 years.  The aim of the present study was to investigate the predictive validity of the OLBI, the Hospital Anxiety Depression Scale (HAD), and of SRH on future LTSA (90 days or more). | Employees in a Swedish County Council,  2,793 only women sample  Thereof, 101  were absent because of musculoskeletal disorders, 77 because of a psychiatric disorder (most often depression and reaction to severe  stress), and 78 were absent because of other diagnoses (including  unknown diagnoses). | Oldenburg Burnout Inventory - OLBI  Exhaustion Disengagement  The Hospital Anxiety Depression Scale (HAD)  Self-Rated Health - SRH | LTSA because of a psychiatric diagnosis  (90 days or more) | **Model 2**  **Adjusted for Age**  OLBI Exhaustion  OR=5.31 (3.02;9.33)  OLBI Disengagement  OR=0.82 (0.48;1.40)  HAD-Depression  OR=1.13 (1.03;1.26)  HAD-Anxiety  OR=1.07 (0.98;1.18)  **Self-Rated Health**  OR=1.74 (1.36;2.24) |
| 21 | Norlund et al. [43]  2011  Sweden | Cohort study  Follow-up 12 months  The aim of this study was to  investigate the impact of psychosocial working conditions and coping strategies at work on change in sick leave level for patients on long-term sick leave due to burnout. | 117 patients on long-term sick-leave due to burnout | Working with people  High demand  Low control  Overtime work more than once a month  High effort reward imbalance  High overcommitment  Use of covert coping towards supervisors  Use of covert coping towards workmates  **Covariates:**  Gender  Age  Rehabilitation program  Education  Sick leave duration | Risk of unchanged or increased level of sick leave at the 12-month follow-up.  -Patients with a lower level of sick leave at t1  than at t0, formed the ‘‘improved’’ group  -Patients with  unchanged or higher level of sick leave at t1 formed the  ‘‘unchanged’’ group | Working with people: OR=0.46 (0.19;1.12)  High demand: OR=0.56 (0.23;1.35)  Low control at work: OR=2.76 (1.10;6.90)  Overtime work more than once a month:  OR=1.09 (0.48;2.48)  High effort reward imbalance:  OR=0.81 (0.33;1.98)  High overcommitment: OR=0.42 (0.18;0.98)  Use of covert coping towards  Supervisors: OR=2.78 (1.17;6.62)  Use of covert coping towards  Workmates: OR=2.58 (1.05;6.34) |
| 22 | Munir et al. [44]  2011  Denmark | Cohort study.  Follow-up 2 years.  DREAM register.  This study sought to examine the influence of protective work factors on long-term sickness absence among employees reporting different levels of depressive symptoms in a representative sample of the Danish workforce. | Year 2000:  5,212 employees  Year 2005:  7,678 employees  Screened for depression by Mental Health Inventory (MHI-5 or MH5)  -3,142 employees were included in both samples | Quality of leadership  Social support  Decision latitude  Covariates:  Sex  Age  Occupational physical activity | Long term SA (sickness spells of at least 3 weeks) | **Group: No depressive symptoms**  **Qual. of leadership**  HR=0.96 / (0.89;1.03)  **Social support**  HR=0.98 (0.91;1.06)  **Decision latitude**  HR=0.91 (0.85;0.97)  **Group: Moderate depressive symptoms**  **Qual. of leadership**  HR=0.88 (0.78;0.98)  **Social support**  HR=1.02 (0.91;1.14)  **Decision latitude**  HR=0.98 (0.88;1.08)  **Group: Severe depressive Symptoms**  **Qual. of leadership**  HR=0.85 (0.67;1.07)  **Social support**  HR=1.07 (0.86;1.33)  **Decision latitude**  HR=1.03 (0.81;1.30) |
| 23 | Hjarsbech et al.[45]  2011  Denmark | Cohort study.  Follow-up 1 year.  The aim of this study  was to investigate how non-clinical and clinical depressive symptoms are prospectively  associated to subsequent LTSA. | 6,985 female employees from the Danish eldercare sector screened for depressive symptoms (Major Depression Inventory) | **Depression**  MDI score (mean, standard deviation)  DSM-IV major depression  ICD-10 unipolar depression  Previous long-term sickness absence  **Covariates:**  **Socio-demographics**  Age  Family status  **Health behaviors**  Smoking  Weekly leisure time physical activity  Body Mass Index  **Occupational group**  Care helpers  Unskilled care helpers  Care assistants  Registered nurses  Therapists/Activity workers  Managers, care  Other functions  Managers, other functions | Risk of long term SA (3 or more weeks) | **Model 4 adjusted for all covariates**  **Depression severity (MDI score)**  **5–9:** HR=1.07 (0.93;1.24)  **10–14:** HR=1.38 (1.15;1.66)  **15–19:** HR=1.54 (1.20;1.98)  **≥20:** HR=1.96 (1.45;2.64)  **Clinical Depression:** HR=2.32 (1.59;3.38)  **DSM-IV algorithm**  **Major depression:** HR=2.05 (1.36;3.08)  **ICD-10 algorithm**  **Clinical Unipolar depression:** HR=2.08 (1.43;3.01) |
| 24 | Hallsten et al. [46]  2011  Sweden | Cohort study.  Follow-up 12 months.  Contingent self-esteem has been assumed to be a risk for burnout-related disorders, and a contingent  self-worth notion of job burnout was applied to study the prospective relationship between job burnout and registered episodes of sickness absence of . 60 consecutive days. | 4,109 employees screened for job burnout and job wornout using the Swedish version of the Maslach Burnout Inventory | Exhaustion-cynicism (from MBI-GS)  Performance-based self-esteem (PBSE)  Job burnout classification  **Covariates:**  Gender  Men  Woman  Age groups  Level of occupational skill  Family status  Chronic disorder  Somatic disorder  Somatic and/or mental disorder  Daily smoking  Previous total sickness absence | Long term SA (60 consecutive days or longer) | ***Job burnout classification***  **Relaxed (ref)**  **Challenged:** OR=0.56 (0.20;1.55)  **Pressured/drained:** OR=1.30 (0.77;2.20)  **Anxious/frustrated:** OR=1.15 (0.68;1.94)  **Job wornout:** OR=0.99 (0.48;2.06)  **Job burnout:** OR=2.05 (1.13;3.70)  **Gender**  **Women:** OR=2.31 (1.50;3.57)  **Age groups**  30–39 years: OR=1.14 (0.65–1.99)  40–49 years: OR=1.19 (0.71;2.01)  50–59 years: OR=1.63 (0.98;2.69)  60–65 years: OR=1.67 (0.84;3.32)  **Level of occupational skill**  **Middle:** OR=0.97 (0.66;1.44)  **Low:** OR=1.25 (0.90;1.75)  **Family status**  **Cohabiting, without children:** OR=0.98 (0.71;1.34)  **Single, without children:** OR=0.79 (0.52;1.20)  **Single, with children:** OR=1.17 (0.76;1.78)  **Chronic disorder**  **Somatic:** OR=1.86 (1.44;2.41)  **Somatic and/or mental:** OR=2.60 (1.51;4.48)  **Daily smoking**  **No (ref)**  **Yes:** OR=1.11 (0.84;1.49)  **Previous total sickness absence**  **1–14 days:** OR=1.72 (1.25;2.36)  **15–59 days:** OR=3.09 (2.10;4.55)  **60 days or more:** OR=4.66 (2.79;7.79) |
| 25 | Catalina-Romero et al. [47]  2012  Spain | Cross-sectional study, with retrospective data collection on previous treatment, sickness absence, or potential confounders.  To identify predictors of long-term sickness absence in  patients with adjustment disorder. | 1,182 subjects with non-work-related sickness absence due to adjustment disorder | **Demographic variables:**  Sex  Age  educational level  Nationality  Marital status  Number of children.  **Work-related variables:** Activity sector  Occupational level (blue collar versus white collar)  Temporary versus  permanent contract  Having more than one job  Income level.  **Clinical and sickness absence-related variables:**  Diagnosis and diagnostic subtypes  Comorbidity  History of sickness absence in the previous 5 years  (Yes/no)  National Insurance contribution status: employee, self-employed, became unemployed during sickness absence | Long term SA (more or equal to six months) | **Comorbidity versus no comorbidity:** OR=2.23 (1.43;3.49)  **Age (years)**  **25–34 versus <25:** OR=2.78 (1.27;6.07)  **35–44 versus <25:** OR=3.70 (1.71;8.00)  **45–54 versus <25:** OR=3.58 (1.60;8.02)  **≥55 versus <25:** OR=6.35 (2.64;15.31)  **Occupational level**  **Blue collar versus white collar:** OR=1.51 / 1.10-2.09 |
| 26 | Salo et al. [48]  2012  Finland | Cohort study.  Follow-up 1 year.  To examine sleep disturbance patterns over time as a risk marker for diagnosis-specific work disability. | 25,639 government employees with diagnostic specific data for sickness absence  In addition to all-cause sickness absence, we analyzed the following main diagnostic groups: malignant tumors (ICD-10 codes C00-C99); mental disorders (F00-F99); diseases of the nervous system (G00-G99), circulatory system (I00-I99), and musculoskeletal system (M00-M99);  and external causes, i.e., injuries and poisonings (S00-T98). | **Sleep history**  No current sleep disturbance  Decreased sleep disturbance  Increased sleep disturbance  Stable moderate sleep disturbance  Stable severe sleep disturbance  **Covariates:**  Age  Sex  Occupational status  Night/shift work  Smoking  High alcohol intake (more than 2010 g/wk)  Obesity (BMI more than 30 kg/m2)  Low physical activity  Physical health problems  Mental health problems  Psychologic distress Anxiety | **Work disability**  Incident diagnosis-specific sickness absence of > 9 days | **Sleep history**  **Mental disorders (918 cases)**  No current sleep disturbance: HR=1.05 (0.82;1.35)  Decreased sleep disturbance: HR=1.37 (1.02;1.83)  Increased sleep disturbance: HR=1.59 (1.32;1.91)  Stable moderate sleep disturbance: HR=1.15 (0.89;1.49)  Stable severe sleep disturbance: HR=1.34 (1.07;1.67) |
| 27 | Gasse et al. [49]  2013  Denmark | Cohort study.  Retrospective 1 year.  To describe patterns and determinants of sick leave among  working Danish antidepressant users. | 25,908 workers starting antidepressant treatment | **Gender**  **Age**  **Transfer payments**  Sick leave in the week before index prescription  Unemployed in the week before index prescription  **Antidepressant at baseline**  SSRI/TCA/SNRI  Other antidepressants  **Marital status**  Single without children  Single with children  Couples without children  Couples with children  Others without children  Others with children  **Education**  Primary school  Secondary school  Vocational education  Higher education, short  Higher education, intermediate  Higher education, long  **Charlson Index**  0/1–2/3 or more  **Musculoskeletal disease**  **Previous history of psychiatric disorders**  Alcohol abuse  Substance abuse  Depression  Anxiety  Schizophrenia  Bipolar disorder  Other psychiatric disease | **Sick-leave**  (more than 2 weeks) | **Stratified: without previous sick-leave (N: 16,197)**  **Gender**  Men: HR=0.78 (0.73; 0.84)  **10-year age-groups**  18–24: HR=0.67 (0.57; 0.78)  25–34: HR=1.19 (1.05; 1.34)  35–44: HR=1.21 (1.07; 1.36)  45–54: HR=1.25 (1.12; 1.40)  55–64: HR=1.0  **Transfer payments**  Unemployed in the week before the  index prescription: HR=1.44 (1.29; 1.60)  **Antidepressant at baseline**  SSRI: HR=1.0  TCA: HR=0.83 (0.74; 0.93)  SNRI: HR=1.05 (0.92; 1.21)  Other antidepressants: HR=0.98 (0.88; 1.09)  **Marital status**  Single without children: HR=1.0  Single with children: HR=1.06 (0.93; 1.22)  Couples without children: HR=1.03 (0.94; 1.13)  Couples with children: HR=1.11 (1.01; 1.22)  Others without children: HR=1.03 (0.88; 1.19)  Others with children: HR=0.94 (0.78; 1.13)  **Education**  Primary school: HR=1.0  Secondary school: HR=0.68 (0.59; 0.78)  Vocational education: HR=1.17 (1.08; 1.27)  Higher education, short: HR=0.93 (0.79; 1.09)  Higher education, intermediate: HR=1.14 (1.02; 1.27)  Higher education, long: HR=0.58 (0.49; 0.68)  **Charlson Index**  0: HR=1.0  1–2: HR=0.99 (0.89; 1.11)  3 or more: HR=1.29 (0.99; 1.69)  **Musculoskeletal disease:** HR=0.90 (0.73; 1.12)  **Previous history of psychiatric disorders**  Alcohol abuse: HR=1.28 (1.04; 1.59)  Substance abuse: HR=0.96 (0.64; 1.43)  Depression: HR=1.00 (0.85; 1.72)  Anxiety: HR=0.66 (0.49; 0.87)  Schizophrenia: HR=0.75 (0.50; 1.11)  Bipolar disorder: HR=1.14 (0.72; 1.83)  Other psychiatric disease: HR=1.08 (0.96; 1.21) |
| 28 | Elovainio et al. [50]  2013  Finland | Cohort study.  Follow-up 1 year.  To assess whether previous psychiatric absenteeism would predict a decline in justice perceptions. | 21,221 participants, public sector employees  (only healthy participants at baseline N=17,641)  N=822 with depression or anxiety disorders | **Procedural Justice**  **Interactional Justice**  (both dimensions were measured by self-report and co-worker assessed)  **Covariates:**  Gender  Age  Type of employment contract  Socioeconomic position  Length of employment  Size of work place  Alcohol use  Smoking  Physical activity  Obesity  Psychological distress in 2004  Chronic disease in 2005  Psychiatric treatment in 2004  Diagnosed mental disorders in 2004  Job strain | Long-term sickness absence (> 9 days) due to diagnosed mental disorders | **(all employees for different subsamples)**  **Model for Procedural Justice**  *Depressive disorders (F32-F34)*  Self-assessed: OR=0.83 (0.70;0.97)  *Anxiety disorders (F40-F42)*  Self-assessed: OR=0.88 (0.75;1.02)  **Model for Interactional Justice**  *Depressive disorders (F32-F34)*: OR=0.87 (0.76;0.99)  *Anxiety disorders (F40-F42):* OR=0.85 (0.74;0.98)  Male: OR=0.63 (0.49-0.81)  Low socioeconomic position: OR=1.15 (1.00-1.32)  Smoking: OR=1.34 (1.10-1.63)  Psychological distress: OR=2.13 (1.80=2.51)  Previous psychiatric treatment: OR=2.76 (2.27-3.35)  Baseline long term sickness absence due to mental disorders: OR=8.29 (6.65-10.34) |
| 29 | Hjarsbech et al. [51]  2013  Denmark | Cohort study, with retrospective data collection (1 year) on previous treatment, sickness absence, or potential confounders.  This study aimed to investigate whether work unit-levels of psychosocial working conditions modify the effect of depressive symptoms on risk of longterm sickness absence (LTSA). | 5,416 employees, Danish female eldercare workers from 309 work units. | One standard deviation increase on the MDI-scale  Quality of leadership  Predictability of work  Work pace  Quantitative demand  **Other covariates:**  Long-term sickness absence  Age  Family status  Smoking  Leisure time physical activity  Body mass index  Working hours  Seniority  Occupational group | Long term SA (three or more consecutive weeks of sickness absence during a 1-year follow-up) | **Depressive symptoms**  **One standard deviation increase on the MDI-scale**  Fully adjusted: RR=1.16 (1.08;1.23)  **Quality of leadership**  Good (ref)  Intermediate: RR=1.04 (0.85;1.27)  Poor: RR=1.19 (0.94;1.49)  **Predictability of work**  Good (ref)  Intermediate: RR=0.92 (0.76;1.11)  Poor: RR=1.12 (0.91;1.38)  **Work pace**  Good (ref)  Intermediate: RR=1.04 (0.86;1.26)  Poor: RR=1.14 (0.91;1.43)  **Quantitative demand**  Good (ref)  Intermediate: RR=0.98 (0.81;1.19)  Poor: RR=1.09 (0.88;1.36) |
| 30 | Hees et al. [52]  2013  The Netherlands | Cohort study  Follow-up 18 months  The present study aimed to examine the temporal and directional relationship between depressive symptoms and various work outcomes in these patients. | 117 long-term sick-listed patients because of MDD | **Demographics**  Gender  Age  Educational level  Marital status  **Clinical characteristics**  Age of onset first depressive episode  More than one depressive episode  Duration of current depressive episode, months  Hamilton Rating Scale for Depression  Co-morbid anxiety disorder  **Work characteristics**  Percentage of absenteeism  Duration of absenteeism, months  Job sector  Work experience in the sector, years  Income, euro  Work productivity  Work Limitations Questionnaire:  Output demands  Time Management demands  Mental/Interpersonal demands | **Absenteeism**  average percentage of sickness absence in the four weeks before an assessment (scores range from 0% to 100% sickness absence) | ***Outcome: percentage of sickness absence***  ***Synchrony of change between depression symptoms***  **HRSD (longitudinal)**  ß=0.02 SE=0.002  (0.01;0.02)  p-value= <0.001 |
| 31 | Vlasveld et al. [53]  2013  The Netherlands | Cross-sectional, with retrospective data collection (6 months) on previous treatment, sickness absence, or potential confounders.  NESDA study.  The aim of this study was to examine the  associations of the Big 5 personality characteristics (neuroticism,  extraversion, openness, agreeableness and conscientiousness)  and locus of control with absenteeism, taking the presence of depressive and anxiety disorders into account. | 1,855 patients with depressive and anxiety disorders and healthy controls  -1,023 (55.1 %) current  depressive or anxiety disorder  - 402 (21.7 %) remitted depressive or anxiety disorder  -430 (23.2 %) healthy controls | **Personality characteristics (big five)**  -neuroticism  -extraversion  -openness  -agreeableness  -conscientiousness  -locus of control  **Diagnosis**  -Current depressive disorder  -Remitted depressive disorder  -Current anxiety disorder  -Remitted anxiety disorder  **Covariates**  -Age  -Gender  -Education  -Marital status  -Number of dependent children  -Number of working hours per week  -Number of somatic conditions  -Job characteristics | Absenteeism  (number of work weeks absent in the last 6 months)  -no absenteeism  -short-term absenteeism (≤2 weeks)  -long-term absenteeism (>2 weeks) | **Psychopathology group**  **0-2 weeks (short-term SA)**  -neuroticism  OR=1.076 (.898; 1.290)  -extraversion  OR=.927 (.788; 1.092)  -openness  OR=1.042 (.886; 1.226)  -agreeableness  OR=1.038 (.886; 1.216)  -conscientiousness  OR=.877 (.750; 1.027)  -locus of control  OR=1.011 (.851; 1.200)  **2 or more weeks (long-term SA)**  -neuroticism  OR=1.458 (1.204; 1.766)  -extraversion  OR=.811 (.684; .960)  -openness  OR=1.063 (.901; 1.255)  -agreeableness  OR=1.118 (.950; 1.315)  -conscientiousness  OR=.747 (.636; .879)  -locus of control  OR=.744 (.625; .885)  **No Psychopathology group**  **0-2 weeks**  -neuroticism  OR=1.747 (1.229; 2.484)  -extraversion  OR=.664 (.471; .935)  -openness  OR=1.002 (.740; 1.358)  -agreeableness  OR=.640 (.464; .884)  -conscientiousness  OR=.713 (.519; .979)  -locus of control  OR=.634 (.433; .928)    **2 or more weeks**  -neuroticism  OR=2.150 (1.314; 3.518)  -extraversion  OR=.517 (.311; .857)  -openness  OR=.599 (.378; .948)  -agreeableness  OR=.804 (.508; 1.275)  -conscientiousness  OR=1.071 (.665; 1.726)  -locus of control  OR=.689 (.400; 1.187) |
| 32 | Hjarsbech et al. [54]  2014  Denmark | Cohort study.  Follow-up: 1 year.  September–December 2008 (T1)  September 2009 (T2)  January 2010 (T3)  May 2010 (T4)  In this study,  we investigated whether organizational justice at work – fairness in resolving conflicts and distributing work –  prevents risk of LTSA among employees with depressive symptoms. | 1,034 participants with depressive symptoms | **Sociodemographic factors**  Gender  Age  Social class  Cohabiting  Having children living at home  **Health-related behavior**  BMI (normal weight)  Smoking habits (never smoked)  Units of weekly alcohol consumption  Hours of weekly physical activity  **Health indicators**  MDI score (0–50)  Physician-diagnosed mental health problem  Number of physician-diagnosed diseases  Psychological treatment (≥1)  Stressful life events (≥1)  **Organizational justice score (0–10)** | Long term SA  (>3 consecutive weeks of SA during follow-up at 12 months) | ***Organizational justice***  **Men**  Intermediate  Model II: RR=0.49 (0.26;0.91)  High  Model II: RR=0.47 (0.20;1.10)  **Women**  Intermediate  Model II: RR=1.29 (0.81;2.06)  High  Model II: RR=0.95 (0.55;1.62) |
| 33 | Janssens et al. [55]  2014  Belgium | Cohort study.  Follow-up 12 months.  The aim was to study the impact of psychosocial risk factors on long-term sickness absence due to mental health problems (LSA-MH) or musculoskeletal disorders (LSA-MSD) in 2983 Belgian middle-aged workers. | Workers from companies or public administrations across Belgium (1,372 men (46%) and 1,611  women (54%).  290 patients with long term SA episodes  -95 due to Mental Health (mainly depression)  -85 due to MSD | Job demands  Job control  Support at work  Job strain  Efforts  Rewards  Effort—Reward imbalance  BulIying  **Covariates:**  Age  Gender  Educational level  Body mass index  Alcohol consumption  Physical activity  Stress outside work  Physical demands at work  Smoking  Baseline depressive symptoms | Long term SA due to mental health problems (at least 15 consecutive days during the last 12 months) | **For group of patients with SA due to CMD**  **Job demands**  Model 2:  OR=0.90 (0.71;1.15); p-value=0.429  **Job control**  Model 2:  OR=0.81 (0.65;1.02); p-value=0.078  **Support at work**  Model 2:  OR=0.83(0.65;1.05); p-value=0.120  **Job strain**  Model 2:  OR=1.06 (0.85;1.33); p-value=0.600  **Efforts**  Model 2:  OR=0.91 (0.71;1.17); p-value=0.465  **Rewards**  Model 2:  OR=0.76 (0.60;0.97); p-value=0.027  **Effort—Reward imbalance**  Model 2:  OR=1.11(0.88;1.39); p-value=0.363  **BulIying**  Model 2:  OR=1.32 (1.06;1.64); p-value=0.012 |
| 34 | Roelen et al. [56]  2014b  The Netherlands | Cohort study.  Follow-up 1 year.  The study aimed to investigate fatigue as prognostic risk marker for identifying working employees at risk of long-term sickness absence (SA). | 633 white collar employees working at an insurance office screened for mental health problems with the Four-Dimensional Symptom Questionnaire (4DSQ). | Total checklist individual strength  Fatigue severity  Reduced concentration  Reduced motivation  Reduced physical activity  **Covariates:**  Age  Gender  Job type | Mental SA  (because SA was medically certiﬁed by an OP in the 3rd or 4th SA week, all SA analyzed in the present study was long-term SA > 3 weeks). | **Men:**  Fatigue severity: OR=1.57 (1.19;2.06)  Reduced concentration: OR=1.75 (1.30;2.35)  Reduced motivation: OR=1.77 (1.34;2.33)  Total CIS: OR=1.97 (1.41;2.75)  **Women:**  Fatigue severity: OR=1.22 (0.88;1.70)  Reduced concentration: OR=1.02 (0.73;1.42)  Reduced motivation: OR=1.17 (0.79;1.74)  Total CIS: OR=1.02 (0.67;1.54) |
| 35 | Roelen et al. [57]  2014a  The Netherlands | Cohort study.  Follow-up 1 year.  This study aimed to investigate mental health symptoms as prognostic risk markers of all-cause and psychiatric sickness  absence (SA) | 1,137 employees working at an insurance office screened for mental health problems with the Four-Dimensional Symptom Questionnaire (4DSQ).  633 (56%) participated in health checks. | Baseline 4DSQ scores for:  Distress  Depression  Anxiety  Somatization | Sickness Absence  (the total number of SA days was accumulated prospectively on the individual level and high SA was defined as 30 SA days during 1-year follow-up). | **Odds-Ratio + Confidence Interval**  4DSQ scale  Distress  Psychiatric SA: OR=1.43 (1.23;1.68)  Depression  Psychiatric SA: OR=1.40 (1.13;1.74)  Anxiety  Psychiatric SA: OR=1.32 (1.07;1.64)  Somatization  Psychiatric SA: OR=1.73 (1.19;2.52) |
| 36 | Smith et al. [58]  2014  Australia | Cohort study.  Follow-up 2 years.  The study aimed to examine if the factors associated with days of absence following a work-related injury  are similar for mental health versus musculoskeletal  (MSK) conditions. | 13,893 claimants for wage-replacement claims  -3,004 mental health claims  -10,899 upper body and back MSK claims  Of the 3,004 mental health claims 11.7 % were for post-traumatic stress, 87.1 % were for  reaction to stressors, which includes depression, anxiety  and other reactions to stress; and 1.2 % were for other mental disorders, which include adjustment disorder, phobias  and psychotic conditions. | **Sociodemographic characteristics**  Age  Gender  Having a previous claim  within the 2 years of the incapacity start date  **Injury characteristics**  number of days between the affliction date (the date recorded as the occurrence of the injury) and the incapacity start date (first day of absence from work)  **Occupational characteristics**  Full-time or part-time before injury  Strength requirements,  Time pressure  Autonomy  **Workplace characteristics**  Size of employer  Industry of workplace | Days of full SA compensation over a two year period. | **Only Claimants for mental health claims**  Age when injured  25–34: ß=0.11 (SE= 0.16 ) p-value= 0.48  35–44: ß=0.24 (SE=0.15) p-value=0.11  45–54: ß=0.28 (SE=0.15) p-value=0.07  55+: ß=0.51 (SE=0.16) p-value=0.001  Gender female: ß=0.03 (SE=0.05) p-value=0.55  Previous claim (yes): ß=0.02 (SE=0.08) p-value=0.76  **Days between injury date and**  **the first day of compensation**  1 day or less:  ß=0.11 (SE=0.07) p-value=0.10  2-7 days = reference  8–30 days:  ß=0.09 (SE=0.08) p-value=0.26  31–90 days:  ß=0.31 (SE=0.10) p-value=0.001  91 or more days:  ß=0.43 (SE=0.08) p-value=<0.001  **Employment type**  Part-time:  ß=-0.01 (SE=0.07) p-value=0.83  **Occupational strength requirements**  Light:  ß=0.12 (SE=0.10) p-value=0.23  Medium:  ß=-0.05 (SE=0.10) p-value=0.60  Heavy:  ß=0.18 (SE=0.10) p-value=0.06  **Occupational time pressure**  50th percentile:  ß=0.13 (SE=0.08) p-value=0.10  75th percentile:  ß=0.23 (SE=0.08) p-value=0.00  100th percentile (high):  ß=0.17 (SE=0.07) p-value=0.03  **Occupational autonomy**  25th percentile (low):  ß=0.11 (SE=0.10) p-value=0.27  50th percentile:  ß=-0.07 (SE=0.07) p-value=0.31  75th percentile:  ß=-0.10 (SE=0.07) p-value=0.16  **Managing employer size**  Small:  ß=0.13 (SE=0.08) p-value=0.11  Large/Gov’t:  ß=-0.23 (SE=0.06) p-value=<0.001  **Industry**  Construction:  ß=0.09 (SE=0.20) p-value=0.66  Wholesale trade:  ß=0.22 (SE=0.15) p-value=0.16  Health care and social assistance:  ß=0.12 (SE=0.12) p-value=0.32  Retail trade accommodation and food services:  ß=0.21 (SE=0.14) p-value=0.15  Transport, postal and warehousing:  ß=0.44 (SE=0.17) p-value=0.01  Public administration and safety:  ß=0.23 (SE=0.12) p-value=0.07  Education and training:  ß=0.42 (SE=0.12) p-value=0.004  Agriculture, forestry, fishing and mining:  ß=-0.72 (SE=0.25) p-value=<0.001  Not elsewhere classified:  ß=0.12 (SE=0.12) p-value=0.29 |
| 37 | Riihimaki et al. [59]  2015  Finland | Cohort study.  Follow-up 5 years.  The study examined the prevalence of predictors for functional and work disability among primary care (PC) patients with depressive disorders in prospective long-term follow-up. | 137 primary care patients with major depressive disorder, dysthymia, subsyndromal MDD and minor depression  -at 18^th^ month follow up: 127  -at 5^th^ year follow-up:  112 | Gender (male)  Age (years)  Chronic medical illness (interfering everyday life)  Social assistance recipient  Time spent in MDE during five years | Duration of SA due to depression (during the last 5 years). | **Outcome: Duration of sick leave**  Gender (male): ß=0.075 (-0.752; 0.902)  Age (years): ß=-0.004 (-0.033; 0.025)  Chronic medical illness  (interfering everyday life): ß=0.935 ( 0.197; 1.674)  Social assistance recipient: ß=1.111 (0.334; 1.888)  Time spent in MDE during five years: ß=0.025 (0.007; 0.042) |
| 38 | Hendriks et al. [60]  2015  The Netherlands | Cohort study.  Follow-up 4 years.  NESDA study.  This longitudinal study aimed to compare long-term work disability and absenteeism between anxiety and depressive disorders focusing on the effects of different course trajectories (remission, recurrence and chronic course) and speciﬁc symptom dimensions (anxiety arousal, avoidance behaviour and depressive mood). | 1632 individuals with a current or lifetime diagnosis of anxiety or depression, and healthy controls(at baseline)  -T1=1420  -T2=1266 | **Baseline psychiatric status**  History of anxiety and/or depressive disorder  History of anxiety and/or depressive disorder x time  Anxiety disorders  Anxiety disorders x time  Depressive disorders  Depressive disorders x time  Comorbid anxiety–depressive disorder  Comorbid anxiety–depressive disorder x time  **Psychiatric course trajectories**  Remission  Recurrence  Chronic course  **Symptom dimensions**  Anxiety arousal  Anxiety arousal x time  Avoidance behavior  Avoidance behavior x time  Depressive mood  Depressive mood x time  **Adjusted for:**  Age, sex, education, number of somatic illnesses,  work contract, work status | Long-term work absenteeism  (the variable work absenteeism was computed by dividing the number of days absent from work during the last six months because of health problems by the number of workdays per week. The maximum number of lost workdays was set at 130 days (5 days per week) for fulltime workers, and in proportion for part-time workers).  No absenteeism, short-term absenteeism (<2 weeks) and long-term absenteeism (>2 weeks). | **Outcome:** Long-term work absenteeism  **Baseline psychiatric status**  History of anxiety and/or depressive disorder  ß=2.16 (SE=.11); p-value=.03  Anxiety disorders  ß=2.15 (SE=.12) p-value=.03  Depressive disorders  ß=5.17 (SE=.13) p-value=<.001  Comorbid anxiety–depressive disorder  ß=4.68 (SE=.14) p-value=< .001  **Psychiatric course trajectories**  Remission: ß=-.59 (SE=.06) p-value=.55  Recurrence: ß=1.42(SE=.08) p-value=.16  Chronic course: ß=2.03 (SE=.06) p-value=.04  **Symptom dimensions**  Anxiety arousal: ß=.60 (SE=.01) p-value=.55  Avoidance behavior:  ß=.25 (SE=.00) p-value=.80  **Depressive mood**  ß=2.15 (SE=.01); p-value=.03 |
| 39 | Roelen et al. [61]  2015  Norway | Cohort study.  Follow-up 2 years.  The study aimed to investigate the predictive value of the 12-item short form (SF-12) for long-term sickness absence (LTSA) because of mental, musculoskeletal,  and other somatic disorders. | 1,381 female nurses, of which 96 developed mental LTSA. | All SF12 factors  Mental component summary (MCS)  Physical component summary (PCS)  Sociodemographics (age, marital status, and care for children at home), lifestyle factors, and work factors from the baseline SUSSH questionnaire were included as covariates in the analyses. | Long-term sickness absence (LTSA), defined as sickness absence episodes lasting more than 16 consecutive days. | **Mental LTSA**  Good = ref.  Poor physical functioning:  OR=0.94 (0.37–2.39)  Poor Physical role limitations:  OR= 1.15 (0.56–2.35)  Poor Bodily pain: OR= 1.33 (0.76–2.34)  **Poor General health**: OR= 2.17 (1.14–4.14)  **Poor Vitality**: OR= 2.24 (1.35–3.72)  **Poor Emotional role limitations**:  OR= 2.85 (1.64–4.95)  **Poor Mental health**: OR= 3.45 (2.25–5.27)  **Poor Social functioning**: OR= 2.42 (1.55–3.78)  **Poor MCS**: OR= 3.15 (2.01–4.93)  Poor PCS: OR= 0.80 (0.36–1.76) |
| 40 | Norder et al. [62]  2015b  The Netherlands | Cohort study.  Follow-up 10 years.  The study investigated the risk of mental health SA among shift compared with day workers. | 5,826 male production workers of a steel plant | Age  Marital status  Work hours per week  Employment duration  Occupational grade (unskilled, semi-skilled, skilled, technician / supervisor) | SA was defined as a temporary leave from work due to injuries and illness. | **Being a shift worker:** HR=1.03 (0.84–1.26)  The risk of mental health SA did not differ between shift and day workers. |
| 41 | Mather et al. [63]  2015  Sweden | Cohort study.  Follow-up 5 years.  To investigate whether psychosocial work environment and health  behaviors are risk factors for sick leave due to mental disorders. | Respondents (n=11,729) that responded to the Study of Twin Adults: Genes and Environment (STAGE).  Sick leave spells due to mental disorders were classified as diagnostic codes F00-F99 in ICD-10. | Age  Gender  Education  Previous sick leave  Self-rated health  Job demands, control, support  Iso-strain (a subgroup among  those with job strain that also had low support)  Health behavior:  Physical activity  Smoking  Alcohol consumption | A binary outcome variable was created with those who had at least one spell of sick leave in a mental diagnosis during the follow-up time (1) and those who had no sick leave during the follow-up (0). | Job demands: OR= 1.41 (1.24–1.60)  Job control: OR= 1.02 (0.90–1.17)  Job support: OR= 1.15 (0.98–1.36)  Low strain=ref.  Passive: OR= 1.02 (0.81–1.28)  Active: OR= 1.30 (1.02–1.67)  Strain: OR= 1.40 (1.12–1.75)  Iso-strain: OR= 1.47 (1.16–1.88)  Alcohol use  Light-moderate/abstainer = ref.  Risk use: OR= 1.09 (0.87–1.35)  Smoking  Nonsmoker=ref.  Current smoker: OR= 1.30 (1.10–1.55)  Physical activity  None/low: OR= 0.91 (0.71–1.16)  Moderate: OR= 0.81 (0.64–1.02)  High: OR= 0.75 (0.60–0.94)  Vigorous=ref.  **Sum score**  Healthy=ref.  Moderately healthy: OR= 1.06 (0.88–1.28)  Unhealthy: OR= 1.34 (1.05–1.69) |
| 42 | Real et al. [64]  2016  Spain | Cohort study.  Follow-up 4 years.  The aim of this study  was to gather data on long-term sickness absence (and relapse) due to mental disorders in Spain. | A cohort of 7112 Spanish patients during the period 2008–2012.  A cohort study of workers registered with a mutual healthcare provider and who had  begun a period of sickness absence due to a mental disorder. | Socio-demographic (age, gender, town/city of residence), clinical (psychiatric diagnosis according to ICD-9), employment-related (type of contract, type of social security contributions, and sector in which employed, coded according to the National Classification of Economic Activities, CNAE 2009), and those related to the sickness absence (duration, reason for end of sick leave). | Long term sickness absence (>60 days). | **Age**  <30: OR=0.54 (0.46–0.64)  30-40: OR= 0.67 (0.59–0.78)  40-50: OR= 0.67 (0.58–0.78)  >50=ref.  **Gender male:** OR= 1.020 (0.965–1.077)  **Type of social security contributions**  General scheme: OR= 0.482 (0.459–0.506)  Self-employed: ref.  Type of contract duration  Permanent: OR= 0.857 (0.793–0.925)  Fixed term: OR= ref.  **Economic activity of employer**  Real estate, construction sector: OR= 1.38 (1.13–1.68)  Older age, severe mental disorders, being self-employed, having a non-permanent contract, and working in the real estate and construction sector were associated with an increased probability of long-term sickness absence. |

**Table 1b: Included articles on prognostic factors for return to work in people with a Common Mental Disorder**

| **No.** | **Author / Year /**  **Country** | **Type of study /**  **Follow-up / Aim** | **Study**  **population** | **Prognostic factors studied** | **Outcome** | **Association estimates (95% CI)** |
| --- | --- | --- | --- | --- | --- | --- |
| 1 | Young & Russell [65]  1995  Australia | Cohort study.  Follow-up at least 1 year.  It was the aim of the current project to identify variables that could predict return-to-work outcomes in a group of teachers who had taken leave for a  work-related stress condition. | 119 teachers who had taken leave for a  work-related stress | Sex  Marital status  Age at start claim  Primary / secondary teacher  The General Health Questionnaire-28 (GHQ).  Time between leave workplace until referral for occupational rehabilitation  Individual attempted to return to work within 505 days. | Return to work | **Discriminant Function Coefficient**  Sex: 0.42609  Age: 0.15824  Type of teacher: 0.32203 |
| 2 | Salkever et al. [66]  2003  United States | Cohort study  Follow-up 3 years  This paper examines the relationship of mental health benefits provided by 116 employers,  to return to work and duration of disability claims for 407 of their employees who were  on long-term disability (LTD) leave for mental disorders. | 407 employees who were on long-term disability (LTD) leave for mental disorders. | Age  Features of benefit -plans  High deductible  Longer preexisting condition Carve out (term not explained)  Health/Mental Health  Benefits and Service  Availability (detailed information in article).  Fringe benefit variables  affecting access to LTD  benefits  **Availability and generosity**  **of LTD and other benefits**  **for employees on LTD**  **Disability management**  **variables** | Return to work  **Duration of the claim**  (time period over which the employee received benefit payments) | **Model Return to work (binary outcome)**  **Demographic characteristics**  AGE  OR=0.9414 <0.001  **Compensation and other benefits (B)**  LT : OR=0.2849 p-value=0.002  LRATIO: OR=0.2116 p-value=0.039  MISSBFT: OR=2.5764 p-value=0.066  RET: OR=0.1606 p-value=0.004  LTDMED: OR=5.9081 p-value=<0.001  WCSUP: OR=3.0636 p-value=0.006  **Disability management practices (M)**  ECONMD: OR=3.6189 p-value=0.01  DMANMBEN: OR=2.14 p-value=0.025  DCONMBEN: OR=0.0674 p-value=<0.001  MGFL: OR=0.371 p-value=0.026  JOBACEE: OR=1.0288 p-value=<0.001  **Disability characteristics**  EXPOSURE: OR=1.0086 p-value=0.005  SQEXPOSURE: OR=0.99999 p-value=0.001  PDEXPOSURE: OR=1.0039 p-value=0.001  **Disability benefits (B)**  STD: OR=0.1822 p-value=<0.001  ELMPRD:OR=0.9852 p-value=<0.001  CONTRIB: OR=0.5425 p-value=0.019  **Health and mental health services**  **coverage and availability (T)**  HIMHDED: OR=0.0247 p-value=<0.001  MHCARVE: OR=0.1853 p-value=0.001  MPREEX: OR=0.8868 p-value=0.001  **Model Duration of the claim**  **Demographic characteristics**  AGE: HR=0.9715 p-value=<.001  **Compensation and other benefits (B)**  EXPDURN: HR=3.0965 p-value=<.001  VARDURYR:  R=0.9978 p-value=.059  YEARDUR: HR=3.0038 p-value=.002  WCSUP: HR=1.494808 p-value=<.001  **Disability management (M)**  ECONMD: HR=0.6595 p-value=.027  MWP: HR=0.7731 p-value=.022  **Disability benefits (B)**  STD: HR=0.4887 p-value=<.001  INFSTD: HR=0.5332 p-value=.009  ELMPRD: HR=0.9959 p-value=<.001  **Health and mental health services availability (T)**  MHCARVE: HR=1.7252 p-value=0.009 |
| 3 | Dewa et al. [67]  2003  Canada | Cohort study.  Follow-up 2 years.  The study aimed to address two questions:  a) prescription of antidepressants in  accordance with published clinical guides associated with better disability outcomes,  b) what is the relationship  between guideline-concordant  antidepressant prescription and length of  disability? | 1,281 workers with depression-related absences from work  for at least 10 consecutive work days prior to their disability leave  Short-term disability  records 1996-1998 were linked for  workers receiving depression-related  short-term disability benefits during that  time. | Gender  Age  Complexity Variables  Guide-line recommended drug use  Type of company | Return to work part-time or full-time.  Length of short-term disability episode for those who returned to work. | **Model for** Return to work part-time or full-time (n=1085)  **Socio-demographic variables**  Female gender: OR=1.41 (0.91;2.20)  Age: OR=0.98 (0.97;0.999)  **Complexity variables**  Number of symptoms  OR=0.83 (0.78;0.89)  Depression only  OR=0.93 (0.69;1.27)  One antidepressant fill only  OR=0.43 (0.16;1.12)  One antidepressant exclusively  OR=0.30 (0.13;0.70)  Switched antidepressants  OR=0.16 (0.069;0.37)  Augmented antidepressants  OR=0.16 (0.069;0.39)  **Guideline-recommended drug use**  Used recommended first-line agent:  OR=1.72 (0.88;3.37)  Used recommended dose:  OR=1.53 (0.94;2.47)  Used within 30 days of short-term benefit start: OR=1.07 (0.68;1.67) |
| 4 | Nieuwenhuijsen  et al. [68]  2004  The Netherlands | Cohort study.  Follow-up 1 year.  Aim of the study: To study supervisory behavior as a predictive factor for return to work. | 81 individuals on sick leave due to mental health problems (depression, anxiety, stress related disorders) for less than six weeks. | Communication with employee  Promoting return to work  Consulting with professionals  Depressive symptoms | **Time to full return to work**  (number of days between the first day of sickness absence and the first  day of full return to work). | **Full return to work**  Communication with employee: HR= 3.5 (1.4; 8.9)  Depressive symptoms: HR= 1.5 (0.6; 3.8)  Communication x depressive: HR= 0.3 (0.1; 0.9)  Promoting return to work: HR= 1.1 (0.4; 2.8)  Depressive symptoms: HR= 0.7 (0.4; 1.2)  Promoting return to work x depressive: HR= 0.6 (0.2; 2.2)  Consulting with professionals: HR= 0.4 (0.2; 0.9)  Depressive symptoms: HR= 0.4 (0.2; 0.9)  Consulting x depressive: HR= 2.0 (0.7; 5.6) |
| 5 | Nieuwenhuijsen et al. [69]  2006  The Netherlands | Cohort study.  Follow-up 1 year. | 188 employees on SL with CMD | **Disorder related factors**  Diagnosis, anxiety or depression  Severity of depressive symptoms, cutoff score 12  Cause of CMD, work-related  Pre-baseline duration of symptoms (3 or more months)  Pre-baseline days out of SA  **Personal factors**  Age, 50 or more years  Gender  Marital Status  Educational level, medium or high  Recovery expectation, (longer than 3 months)  **Environmental factors**  Job demands  Supervisory support  Co-worker support | Time until full RTW (working as many hours as before the onset of the sickness absence, for at least 1 week). | **Age, 50 or more years:** HR=0.5 (0.3;0.8)  **Patient’s recovery expectation:** HR=0.5 (0.3;0.8)  **Educational level, medium or high:** HR=0.5 (0.3;0.8)  **Diagnosis, anxiety disorder or depression:** HR=0.7 (0.4;0.9) |
| 6 | Engström & Janson [70]  2007  Sweden | Cohort study.  Follow-up 3 years.  The study aimed to analyze factors influencing chances of returning to work after long-term sickness absence with a stress-related psychiatric diagnosis. | 911 individuals with stress-related psychiatric disorders (i.e., stress, burn-out syndrome, chronic fatigue etc.) | Gender  Employer  Occupation  Age  Previous sickness  Pain diagnosis | RTW after long Term SA (absence spells of more than 28 days) | No confidence intervals were provided.  **Men**  At 2-year follow-up: OR= 1.219  At 3-year follow-up: OR= 1.637***  **Employer category**  **Municipality elderly care**  At 2-year follow-up: OR= 0.887  At 3-year follow-up: OR= 1.086  **Munic. Children & education**  At 2-year follow-up: OR= 0.917  At 3-year follow-up: OR= 1.386  **Municipality other**  At 2-year follow-up: OR= 1.090  At 3-year follow-up: OR= 1.954*  **County. Caring**  At 2-year follow-up: OR= 0.423*  At 3-year follow-up: OR= 0.983  **County. other**  At 2-year follow-up: OR= 0.726  At 3-year follow-up: OR= 0.958  **Private**  At 2-year follow-up: OR= 0.742  At 3-year follow-up: OR= 0.935  **Self employed**  At 2-year follow-up: OR= 1.399  At 3-year follow-up: OR= 2.008  **Unemployed**  At 2-year follow-up: OR= 0.412**  At 3-year follow-up: OR= 0.468**  **Public other**  **(reference)**  **Occupational category**  **Management**  At 2-year follow-up: OR= 1.020  At 3-year follow-up: OR= 0.903  **Caring**  At 2-year follow-up: OR= 0.821  At 3-year follow-up: OR= 0.976  **Education**  At 2-year follow-up: OR= 0.877  At 3-year follow-up: OR= 0.985  **Service**  At 2-year follow-up: OR= 0.876  At 3-year follow-up: OR= 0.808  **Other**  **(reference)**  **Age**  **16 – 29**  At 2-year follow-up: OR= 14.912***  At 3-year follow-up: OR= 16.961***  **30 – 39**  At 2-year follow-up: OR= 13.985***  At 3-year follow-up: OR= 17.344***  **40 – 49**  At 2-year follow-up:OR= 7.130***  At 3-year follow-up: OR= 7.956***  **50 – 59**  At 2-year follow-up: 3.800***  At 3-year follow-up: 4.807***  **60 – 64**  **(reference)**  **Previous sickness absence**  At 2-year follow-up: OR= 0.613***  At 3-year follow-up: OR= 0.617***  **Co morbid Pain diagnosis**  At 2-year follow-up: OR= 0.489***  At 3-year follow-up: OR= 0.576*** |
| 7 | Brouwers et al. [71]  2009  The Netherlands | Cohort study.  Follow-up at 3 and 6 months.  This study aimed to investigate which factors predict return to work (RTW) after 3 and 6 months in employees  sick-listed due to minor mental disorders. | 194 subjects at the start of sick leave due to minor mental disorders (generalised anxiety disorder, mild major depressive disorder, or no mood or anxiety disorder according to the CIDI) | **Socio-demographic characteristics**  Gender  Age  Level of education  Has partner  Has children aged 0–11  Has children aged 12–18  Works part time (32 or less hours)  Chronic somatic disease  **Problem related characteristics**  Previous mental problems  Onset symptoms [more than 3 months before T0  Onset sick leave 3 weeks or more before T0  Own prediction RTW of less than 6 weeks  Attributes cause of absenteeism to:  Work  Health problems  Relationship with partner  Family  Relatives  Financial problems  Stressful life event  Other problems  **Health care use T0 and T1**  Contact with GP past 4 weeks  Contact with OP past 4 weeks  Contact medical specialist past 4 weeks  Contact mental health professional past 4 weeks  Contact physical therapist past 4 weeks  Contact complementary healer  Benzodiazepam use  Antidepressant use  **4DSQ scores (T0)**  Distress  Depression  Anxiety  Somatization | Full return to work (yes versus no). | **Prediction of RTW at T1 using baseline data**  Problems started more than 3 months before T0:  OR=0.395 (0.204–0.765)  Own prediction of sick leave duration\6 weeks:  OR=2.278 (1.031–5.035)  Contact OP in past 4 weeks:  OR=0.489 (0.251–0.954)  4DSQ somatization score at T0:  OR=0.926 (0.876–0.980)  **Prediction of RTW at T2 using baseline data**  Treatment condition  (GP = 0; social work = 1):  OR=0.573 (0.264–1.244)  Problems started more than 3 months before T0:  OR=0.275 (0.131–0.577)  Absenteeism more than 3 weeks at T0:  OR=0.384 (0.185–0.799)  Attributes cause of absenteeism to family problems:  OR=0.204 (0.052–0.808)  Interaction effect: attribution cause of absenteeism to family problems x treatment condition:  OR=10.440 (1.550–70.341)  Contact physical therapist in past 4 weeks:  OR=5.542 (0.502–61.206)  Interaction effect: contact physical therapist in past 4 weeks x treatment condition:  OR=0.050 (0.003–0.971)  4DSQ anxiety score at T0:  OR=0.902 (0.826–0.985)  **Prediction of RTW at T2 in those who had not yet resumed work at T1**  Absenteeism more than 3 weeks at T0:  OR=0.335 (0.137–0.818)  4DSQ depression score at T1:  OR=0.738 (0.572–0.953) |
| 8 | Brouwer et al. [72]  2010  The Netherlands | Cohort study.  Follow-up 1 year.  The aim of the present study was to conduct subgroup-analyses in a prospective cohort of workers on long-term sickness absence to investigate whether  associations between perceived work attitude, self- efficacy and perceived social support and time to RTW differ  across different health conditions. | 926 workers on sickness absence (6–12 weeks),  245 with mental health condition (stress, depression, burnout) | **Age**  **Sex**  **Educational level**  Very low  Low  Medium  High  **Time to identification by OHS (in days, SD)**  **Intensity of conditions (0–100)**  **Full return to work at 10 month follow-up**  **Work attitude**  **Social support**  **Self-efficacy**  Willingness to expend effort in completing a behavior  Willingness to initiate behavior  Persistence in the face of adversity | Time to RTW (deﬁned as the time between sickness absence identiﬁcation by the OHS and ﬁrst full RTW).  Employees were followed until the tenth month after listing sick**.**  Workers had to indicate their current work status: full RTW, partial RTW or being on full sick leave. Full RTW was deﬁned as working the same number of hours as in the initial work contract. | **Mental health subgroup**  Sex (female): HR=1.10 (0.79;1.52) p-value=0.58  **Age (4 categories) years**  35–44: HR=0.92 (0.73;1.49) p-value=0.75  45–54: HR=1.07 (0.68;1.70) p-value=0.77  55–64: HR=1.09 (0.62;1.91) p-value=0.77  **Educational level (4 categories)**  Low: HR=2.57 (1.34;4.94) p-value=0.01  Medium: HR=1.60 (0.85;2.47) p-value=0.04  High: HR=1.60 (1.10;2.33) p-value=0.01  **Duration to identification by the OHS (4 categories) days**  43–54: HR=1.03 (0.68;1.57) p-value=0.88  55–70: HR=0.73 (0.46;1.14) p-value=0.17  ≥ 71: HR=0.90 (0.57;1.41) p-value=0.65  Intensity of conditions: HR=0.99 (0.98;1.00) p-value=0.16  Work attitude (high): HR=0.94 (0.68;1.29) p-value=0.69  Social support (high): HR=0.80 (0.58;1.11) p-value=0.18  ***Self-efficacy***  **Willingness to expend effort in**  **completing a behavior:**  HR=1.49 (1.01;2.18) p-value=0.04  Multivariate: HR=1.60 (1.07;2.40) p-value=0.02  **Willingness to initiate behavior:**  HR=1.19 (0.85;1.67) p-value=0.32  **Persistence in the face of adversity:** HR=1.16 (0.83;1.62) p-value=0.39 |
| 9 | Hoedeman et al. [73]  2010  The Netherlands | Cohort study.  Follow-up 2 years.  Retrospective data 1 year  Secondary objective of the study was to evaluate determinants of the duration of sickness absence in employees with high levels of somatic symptom severity (HLSSS). | 489 sick-listed employees with CMD. | PHQ +15  Age (per year)  Autochthon  PHQ-9  Whitely Index  Distress (per 8 on 4DSQ)  PHQ other anxiety disorder  PHQ panic disorder  PHQ other anxiety disorder  Group practice  Employees attribution | Duration until complete RTW (as duration of sickness absence)  Sickness absence periods (duration in days) | **Model 2**  **High levels of somatic symptom severity (PHQ+):** HR=0.65 (0.46;0.93)  **Age (per year):** HR=0.98 (0.97;0.99)  **Health Anxiety (Whitely):** HR=0.94 (0.91;0.98)  **Group practice:**  1: HR=1.00  2: HR=1.64 (1.14;2.36)  3: HR=1.03 (0.74;1.43)  4: HR=1.43 (1.03;2.00)  5: HR=1.50 (1.03;2.18) |
| 10 | Nielsen et al. [74]  2011  Denmark | Cohort study.  Follow-up 52 weeks.  The study aimed to identify health-, personal- and work-related factors  predictive of return to work (RTW) in employees sick-listed due to common mental health problems, such as, stress, depression, burnout, and anxiety. | 644 employees from the Municipality of  Copenhagen who applied for sickness benefits due to CMD. | Gender  Age (in years)  RTW expectancy  Prior absence with MHP  Occupation:  Research, art and technical work  Management  Administration  Trade  Service  Manual workers  Health care  Social work  Education  Self-reported reason for absence | Time to RTW (was measured in weeks starting from the first day of absence until the first week of RTW)  (~ Duration of SA) | **Gender (women)**  Multivariate: HR=1.02 (0.80;1.31)  **Age (in years) (50 or more)**  Multivariate: HR=1.17 (0.91;1.50)  **RTW expectancy (yes)**  Multivariate: HR=1.25 (0.97;1.61)  Fitted model: HR=1.27 (1.01;1.61)  **Prior absence with MHP (no)**  Multivariate: HR=1.23 (0.94;1.60)  Fitted model: HR=1.29 (1.01;1.64)  ***Self-reported reason for absence***  **Depression**  Multivariate: HR=0.73 (0.58;0.93)  Fitted model: HR=0.76 (0.61;0.94)  **Other MHP**  Multivariate: HR=0.53 (0.29;0.99)  Fitted model: HR=0.56 (0.31;1.00)  **Unspecific MHP**  Multivariate: HR=0.80 (0.56;1.14)  Fitted model: HR=0.87 (0.62;1.20)  ***Occupation***  **Management:** Multivariate: HR=0.83 (0.35;1.93)  **Administration:** Multivariate: HR=0.97 (0.68;1.39)  **Trade:** Multivariate: HR=0.70 (0.43;1.15)  **Service:** Multivariate: HR=0.96 (0.64;1.45)  **Manual workers:** Multivariate: HR=0.94 (0.60;1.48)  **Health care:** Multivariate: HR=1.44 (0.94;2.21) |
| 11 | Virtanen et al. [75]  2011  Finland | Cohort study.  Follow-up 6 years.  The study aimed to examine the associations between socio-economic position (SEP) and the onset of psychiatric work disability, return to work and recurrence of disability. | Register data on 141 917 public-sector employees in Finland. | Socio-economic position (SEP)  Controlled for age, sex, geographic area and employer. | RTW after long-term  sickness absence (≥90 days) | **Depression**  Manual=1  Lower non-manual: HR= 1.25 (1.10 to 1.42)  Higher non-manual: HR= 1.45 (1.26 to 1.67)  **Anxiety disorders**  Manual=1  Lower non-manual: HR= 1.35 (0.96 to 1.90)  Higher non-manual: HR= 1.31 (0.87 to 1.97)  **Severe stress and adjustment disorders**  Manual=1  Lower non-manual: HR= 1.04 (0.72 to 1.52)  Higher non-manual: HR= 0.85 (0.56 to 1.28) |
| 12 | Hees et al. [76]  2012  The Netherlands | Cohort study.  Follow-up 18 months.  The study aimed to identify health-, personal- and work-related factors  predictive of return to work (RTW) in employees sick-listed due to common mental health problems, such as, stress, depression, burnout, and anxiety. | 117 participants with MDD. | **Demographic**  Age, less than 50  Educational level  **Diagnostic**  Depression severity (HDRS and IDS-SR)  Comorbid anxiety  MOS SF-36  Physical functioning  Bodily pain  General health perceptions  Social functioning  Mental health  Vitality  **Work-related**  Percentage of SA  Recovery expectations  Income, less than median  Work motivation  WQL  Physical work limitations  Time work limitations  Output  Mental/interpersonal  VBBA  Job satisfaction  Emotional workload  Relationship with colleagues  Relationship with supervisor  **Personality-related**  UCL  Expression of emotions  NEO-FFI  Neuroticism  Openness  Altruism  Conscientiousness | Long term full RTW (working full number of contract hours for at least 4 weeks). | **Outcome: Long term full RTW**  ***Univariate analysis***  **Demographic**  Age, less than 50  OR=0.23 (0.04;1.32)  Educational level  OR=1.53 (0.93;2.25)  ***Multiple Regression Model***  **Diagnostic**  Depression severity (IDS-SR)  OR=0.92 (0.87;0.97)  Comorbid anxiety  OR=0.21 (0.05;0.84)  **Work-related**  Work motivation  OR=1.87 (1.18;2.96)  Conscientiousness (NEO-FFI )  OR=1.10 (1.02;1.18) |
| 13 | Nielsen et al. [77]  2012  Denmark | Cohort study.  Follow-up 52 weeks.  The study aimed to identify health-, personal- and work-related factors  predictive of return to work (RTW) in employees sick-listed due to common mental health problems, such as, stress, depression, burnout, and anxiety. | 205 employees who applied for sickness absence benefits due to a self-reported MHP to the Job Centre Copenhagen,  a municipal welfare department. | Age  Self-rated health  Shoulder/neck pain  Low back pain  DSM-IV Depression (no)  Gender (women)  Education (low)  Cohabitation (no)  Employment at baseline  Size of workplace  Sector | Time to RTW **(**was measured from the first day of absence until RTW or censoring. Time to RTW was defined as no longer receiving sickness absence or unemployment benefits). | **Univariate analysis**  Age (one year increase): HR=1.01 (0.98;1.02)  Self-rated health (one point increase): HR=1.19 (1.04;1.35)  Shoulder/neck pain (one point increase): HR=0.94 (0.89;1.00)  Low back pain (one point increase): HR=0.94 (0.88;1.00)  DSM-IV Depression (no): HR=0.59 (0.43;0.80)  Gender (women): HR=0.82 (0.56;1.19)  Education (low): HR=0.94 (0.67;1.31)  Cohabitation (no): HR=1.06 (0.79;1.43)  Size of workplace: HR=1.33 (0.93;1.90)  Sector:  Private HR=0.65 (0.44;0.95)  Municipal HR=0.62 (0.41;0.94)  **Multivariate analysis**  Self-rated health: HR=1.18 (1.03;1.34)  DSM-IV Depression: HR=0.61 (0.45;0.84)  Sector:  Governmental (ref.)  Private HR=0.65 (0.44;0.96)  Municipal HR=0.62 (0.41;0.94) |
| 14 | Sampere et al. [78]  2012  Spain | Cohort study  Follow-up 24 months (max.)  The study examines the associations between time to RTW and sick-listed worker perceptions of their health status, work ability, RTW expectations and time required to RTW, self-efficacy and self-perceived connection between health and job in employees on long-term non-work-related sickness absence. | 663 workers with a sick-leave episode were followed until their sick leave episode ended.  Cohort participants included workers with a current non work-related sick leave episode exceeding 15 days (long-term non work-related sick leave episode).  Psychoses, neurotic disorders and mental retardation (ICD-9 codes 290-319) | **Sociodemographic variables**  Age  Sex  Co-habitation status  **Occupational factors** Physical activity  job insecurity | Time to return to work (was calculated from the difference in days between the date of the end and the start of sick leave episode, plus one). | **Model** (adjusted for covariates)  **General health status**  Poor: HR=0.96 (0.55;1.69)  **Work ability**  Moderately reduced  HR=4.14 (0.87;19.72)  Very or extremely reduced  HR=2.93 (0.62;13.92)  **Estimated Time required to RTW**  <1 month = reference  1–3 months: HR=0.34 (0.16;0.69)  3 months: HR=0.41 (0.12;1.35)  I will never be able to: HR=0.12 (0.03;0.43)  I don’t know: HR=0.39 (0.23;0.67)  **Relation between health and job**  No=reference  Yes, partially: HR=0.43 (0.23;0.81)  Yes, completely: HR=0.41 (0.20;0.82)  **General self-efficacy**  High=reference  Moderate: HR=0.69 (0.28;1.71)  Low: HR=1.03 (0.57;1.85) |
| 15 | Soegaard [79]  2012  Denmark | Retrospective cohort study.  Follow-up 1 year.  This study aimed to estimate (1) the frequencies of CMD, (2) the predictors of undetected CMD, and (3) the rate of return to work among sick listed individuals without a psychiatric disorder, who are registered on long-term sickness absence (LSA) | 831 individuals with sickness absence screened for Common Mental Disorders-Screening Questionnaire (CMD-SQ) | Depression  Anxiety  Somatoform disorder  White collar/civil servant  Somatoform ∗ White collar/civil servant  Employment-Skilled worker  Age 60 years +  Unemployed | Return to work rate analyzed by means of survival methods by which observation period was defined as the period from the ﬁrst day of entering LSA until the payment of sickness beneﬁts was stopped. | **Depression:** HR=0.59 (0.54;0.64)  **Anxiety:** HR=1.50 (1.29;1.74)  **Somatoform disorder:** HR=1.43 (1.18;1.74)  **White collar/civil servant:** HR=1.40 (1.36;1.44)  **Somatoform ∗ White collar/civil servant:** HR=0.07 (0.03;0.19)  **Employment-Skilled worker:** HR=2.25 (2.11;2.39)  **Age 60 years +:** HR=1.94 (1.77;2.13)  **Unemployed:** HR=0.33 (0.28;0.38) |
| 16 | Vemer et al. [80]  2013  The Netherlands | Cohort study.  Follow-up until RTW (on average 34 weeks).  This study aimed to assess the added value of  health-related quality of life (HRQoL) and severity of depression to predict the time to RTW. | 122 workers on sickness absence (between 4 and 12 weeks) due to major depression disorder (MDD). | **Demographic**  Female  Age  Living condition  Education  **Job related**  decision latitude  skill discretion  decision authority  psychological job demands  physical job demands  physical exertion  physical isometric loads  social support  co-worker support supervisor support  **Health related**  Severity of depressive  symptoms (PHQ-9)  Somatization  Health related quality of life (EQ-5D and SF-6D) | Duration until full RTW, starting from the moment of baseline measurement.  Full RTW was defined as the first full RTW with equal earnings, lasting for at least 4 weeks. | **Personal variables**  Female  Full model: ß=0.868***  Age (in years)  Full model: ß=0.028***  Living with adult partner, no children  Full model: ß=0.433**  **Job-related variables**  Holds a management function  Full model: ß=-0.635***  Work week ≥ 36 hours  Full model: ß=0.686***  More decision latitude (JCQ)  Full model: ß=0.018**  More social support (JCQ)  Full model: ß=-0.075**  Intervention (collaborative care)  Full model: ß=-0.098 |
| 17 | Nieuwenhuijsen et al. [81]  2013  The Netherlands | Cohort study.  Follow-up 1 year.  This study aims to assess the relative value of RTW self-efﬁcacy (RTW-SE) and RTW expectation in predicting actual RTW | 179 workers on sick leave due to common mental disorders between two and 8 weeks | Self-Efﬁcacy for Return to Work (RTW-SE)  RTW Expectation | Time to full return to work. | Model 2:  **RTW-Self Efficacy** log rank test: (Chi2 = 17.8 (df = 1), P<0.000)  RTW expectations log rank test: not significant |
| 18 | Ekberg et al. [82]  2015  Sweden | Cohort study.  Follow-up 1 year.  The study aims to identify individual and workplace factors associated with early return to work (RTW) defined as within 3 months and factors  associated with later RTW between 3 and 12 months after being sick-listed. | 533 sick-listed individuals with common mental  diagnoses (depression, anxiety, stress, burnout and other CMD) | **Sex**  Female/Male  **Education**  Compulsory school  Upper secondary school  University  Financial Strain  **Occupational code**  White/Pink/Blue  **Self-rated health**  EQ-5D/EQ-VAS  Burnout  Depression (Zung SD)  Pain-VAS  Karolinska Sleep Questionnaire  Functional Rating Index, FRI  Work Ability Index, WAI  **Personal Resources**  Mastery  Availability of Attachment  Availability of social integration index  Symptom satisfaction  Expectation of RTW  Expectations of treatment  **Self-rated work conditions**  Effort-reward index  Over commitment  Justice  Physical load  Exit behavior | RTW (within 3 months).  RTW (between 3 and 12 months)  Number of calendar days of absence (full- or part-time) until the first sustainable (>4 weeks) RTW | **Outcome: RTW <3 months**  Nine-years compulsory education  HR=2.40 (1.46;3.95)  College education  HR=1.14 (0.83;1.57)  Work Ability Index, WAI  HR=1.08 (1.05;1.10)  Interactional justice  HR=0.83 (0.71-0.96)  Positive expectations of treatment  HR=1.50 (1.04;2.16)  **Outcome: RTW 3-12 months**  Exit  HR=1.16 (1.01;1.33)  Need for reduced demands  HR=1.64 (1.08;2.50) |
| 19 | Netterstrøm et al. [83]  2015  Denmark | Cohort study.  Retrospective data 1 and 3 years.  The aim of this study was to assess the prognostic factors of return to work (RTW) after one and three years among people on sick-leave due to occupational stress. | 223 completers on sick-leave, who participated in a stress treatment program. | Self-reported psychosocial work environment, life events during the past year, severity of the condition, occupational position, employment sector, marital status, and medication | RTW after 1 year (yes versus no) | **Multivariable analysis, Model 1 (adjustment for age, gender, and occupational position)**  **Low decision authority:** OR=0.982 (0.970–0.999)  **Bullying:** OR=0.715 (0.513–0.994)  **Workability index:** OR=1.220 (1.038–1.434)  **Full-time sick-leave at baseline:** OR=0.431 (0.222–0.830)  Demands and social support from leaders as well as colleagues were significantly associated with RTW after adjustment for age, gender, and occupational position whereas the predictability and rewards  were only borderline significant (0.1 > 𝑃 > 0.05) (data not shown). |
| 20 | Norder et al. [84]  2016  The Netherlands | Cohort study.  Follow-up 1 year.  The objective of the present study was  to validate an existing prediction rule for predictions of the duration of sickness  absence due to common mental disorders (CMDs) and investigate the added value of work-related factors. | 596 employees working in companies with a sickness absence insurance who reported sick with CMDs in the period from  September 2013 to April 2014. | Age  Gender  Educational level  Recovery expectations  Type of CMD | Time to the day of full return to work (i.e. working the same number of hours per week as before CMD sickness absence)  Sickness absence (no = 0, yes = 1) at 3, 6 and 12 months after reporting sick with CMDs. | **Variety in work** increased the Integrated Discrimination Improvement (IDI): 2.93 (0.00; 5.77), discrimination improved to AUCs of 0.74 (95 % CI 0.63–0.85) and 0.62 (95 % CI 0.52–0.72) for CMD sickness  absence at 3 and 6 months, respectively. |
| 21 | Prang et al. [85]  2016  Australia | Retrospective cohort study.  Follow-up 2 years.  The aims of this study were to describe predictors of sustained return to work (RTW) among a cohort of workers with compensated work-related mental health conditions (MHCs); and to examine predictors of subsequent absences due to the same condition. | 8,358 workers | Age, gender  Occupational skill level  Previous claims  Injury mechanism  Workplace size  Industry group  Mental Health service consultation | The primary outcome was time until a ﬁrst sustained RTW during the two year follow-up period as measured in days.  First sustained RTW was deﬁned as return to pre-injury hours denoted by a gap in wage replacement payments of 30 days or longer following the date of the ﬁrst incapacity related to the MHC.  Sustained RTW, based on compensation  claims data. | HR <1 reﬂects a longer time to RTW  **Age**  15 to 25 years=ref.  26 to 35 years: HR=0.79 (0.71–0.88)  36 to 45 years: HR=0.69 (0.62–0.77)  46 to 55 years: HR=0.65 (0.58–0.72)  56 plus: HR=0.64 (0.57–0.72)  **Gender female:** HR=0.93 (0.88–0.98)  **Previous claims**: HR=0.94 (0.88–0.99)  **Injury mechanism**  Harassment/bullying= Ref  Work pressure: HR= 1.03 (0.97–1.09)  Other mental stress factors:  HR= 1.04 (0.98–1.11)  Assault/workplace violence:  HR= 1.08 (0.98–1.18)  Exposure to a traumatic event:  HR= 1.10 (1.00–1.22)  Other mechanism of injury:  HR=1.33 (1.20–1.47)  **Occupational skill level**  Bachelor degree/5 year training =Ref  Diploma/3 year training:  HR= 1.06 (0.99–1.13)  Certiﬁcate/3 year training:  HR= 1.13 (1.02–1.25)  Certiﬁcate/1 year training:  HR= 1.12 (1.05–1.20)  No formal training: HR= 1.15 (1.05–1.26)  **Workplace size**  Small: HR= 0.81 (0.73–0.89)  Medium: HR= 0.97 (0.90–1.05)  Large: HR= 1.15 (1.07–1.23)  Government=ref.  **Industry group**  Mental Health service consultation  Psychologist (Yes/No): HR= 0.83 (0.78–0.88) <0.01  Psychiatrist (Yes/No): HR=0.88 (0.83–0.93) |

**Table 1c: Included articles on prognostic factors for recurrent sickness absence in people with a Common Mental Disorder**

| **No.** | **Author / Year /**  **Country** | **Type of study /**  **Follow-up / Aim** | **Study**  **population** | **Prognostic factors studied** | **Outcome** | **Association estimates (95% CI)** |
| --- | --- | --- | --- | --- | --- | --- |
| 1 | Koopmans et al. [86]  2010  The Netherlands | Cohort study.  2001-2007.  The aim of this study was to investigate the recurrence of sickness absence due to CMDs  including distress, adjustment disorders, depressive disorders and anxiety disorders, according to age, in male and female employees in the Netherlands. | 52,629 workers in the Dutch post and telecommunication companies, with a sickness absence due to CMD | Psychiatric diagnosis  CMD  Stress  Gender  Age | Recurrence of SA | Women < 35 years  Women 35-44 years  No differences in age groups in men. |
| 2 | Roelen et al. [87]  2010  The Netherlands | Dynamic cohort study  (from 2001 to 2007).  Study aim: To investigate the recurrence of sickness absence according to diagnosis. | 6,211 employees working for the Dutch Post and Telecom, with SA due to CMD. | Gender  Age  Marital status  Socioeconomic status  Employment (Full-time or  Part-time)  Duration of employment  Salary scale | Recurrent SA (due to mental disorders).  Episodes of certiﬁed sickness absence starting more than 28 days after full return to work with equal earnings were regarded as recurrences. | **Gender**  Male: RR=1  Female: RR=1.00 (0.93;1.09)  **Age**  Less than 35 years: RR=1  35–44 years: RR=0.92 (0.85;1.01)  45–54 years: RR=0.91 (0.82;1.00)  55 or more years: RR=1.05 (0.92;1.20)  **Marital status**  Unmarried: RR=1  Married: RR=0.95 (0.89;1.01)  **Socioeconomic status**  Low: RR=1  Lower average: RR=1.00 (0.92;1.09)  Upper average: RR=1.02 (0.94;1.11)  High: RR=1.01 (0.93;1.09)  **Employment**  Full-time: RR=1  Part-time: RR=1.03 (0.94;1.12)  **Duration of employment**  Less than 5 years: RR=1  5–9 years: RR=0.72 (0.65;0.79)  10–14 years: RR=0.81 (0.73;0.90)  15–19 years: RR=0.81 (0.73;0.91)  20 or more years: RR=0.81 (0.74;0.89)  **Salary scale**  1 and 2: RR=1  3: RR=0.92 (0.82;1.03)  4 and 5: RR=0.88 (0.78;0.99)  6 and 7: RR=0.87 (0.78;0.98)  8 or more: RR=0.82 (0.72;0.93) |
| 3 | Koopmans et al. [88]  2011  The Netherlands | Cohort study.  Follow-up 7 years.  Study aim: To determine which determinants are related to the recurrence of  sickness absence due to CMDs. | 9,904 employees working in the Dutch Post or Telecom  company, with a sickness absence due to CMD | CMD  Distress symptoms  Adjustment disorder  Depressive symptoms  Anxiety symptoms  Other psychiatric disorders  Age  Marital status  Salary scale  Full-time  Tenure  Telecom or Post | Recurrent SA  A recurrence is defined as the start of a new episode of sickness absence due to CMDs after a recovery period of at least 28 days. | ***Initial episode***  **Adjustment disorder**  Men: RR=1.03 (0.91;1.16)  Women: RR=1.15 (0.99;1.33)  **Depressive symptoms**  Men: RR=1.30 (1.07;1.59)  Women: RR=1.24 (0.99;1.54)  **Anxiety symptoms**  Men: RR=1.00 (0.74;1.35)  Women: RR=1.16 (0.81;1.67)  **Other psychiatric disorders**  Men: RR=1.19 (1.00;1.42)  Women: RR=1.26 (1.03;1.53)  **Age**  <35 years  Men: RR=1.12 (0.86;1.47)  Women: RR=1.72 (1.18;2.51)  35–44 years  Men: RR=1.22 (0.99;1.51)  Women: RR=1.61 (1.12;2.30)  45–54 years  Men: RR=1.23 (1.02;1.50)  Women: RR=1.39 (0.97;2.00)  **Unmarried**  Men: RR=0.92 (0.82;1.04)  Women: RR=0.82 (0.72;0.94)  **Salary scale 1–2**  Men: RR=1.39 (1.04;1.86)  Women: RR=1.60 (1.17;2.19)  Salary scale 3  Men: RR=1.67 (1.36;2.05)  Women: RR=1.91 (1.37;2.65)  Salary scale 4–5  Men: RR=1.28 (1.04;1.58)  Women: RR=1.29 (0.96;1.73)  Salary scale 6–7  Men: RR=1.36 (1.12;1.65)  Women: RR=1.16 (0.87;1.53)  **Full-time job**  Men: RR=1.09 (0.93;1.28)  Women: RR=0.99 (0.82;1.19)  **Tenure**  <5 years  Men: RR=1.02 (0.85;1.23)  Women: RR=1.29 (1.01;1.66)  5–9 years  Men: RR=1.03 (0.84;1.25)  Women: RR=1.03 (0.79;1.34)  10–14 years  Men: RR=1.10 (0.91;1.34)  Women: RR=0.99 (0.77;1.27)  15–19 years  Men: RR=0.99 (0.82;1.20)  Women: RR=1.08 (0.84;1.41)  **Telecom**  Men: RR=1.33 (1.13;1.57)  Women: RR=1.25 (1.02;1.53) |
| 4 | Virtanen et al. [75]  2011  Finland | Cohort study.  Follow-up 6 years.  The study aimed to examine the associations between socio-economic position (SEP) and the onset of psychiatric work disability, return to work and recurrence of disability. | Register data on 141,917 public-sector employees in Finland. | Socio-economic position (SEP)  Controlled for age, sex, geographic area and employer. | Recurrence of SA (≥90 days). | Higher non-manual =1  Lower non-manual: HR= 1.15 (0.96 to 1.37)  Manual: HR= 1.25 (1.02 to 1.53) |
| 5 | Ervasti et al. [89]  2014  Finland | A cohort study.  Observation period 2005–2011  The specific aims of the study were to investigate the extent to which comorbid other psychiatric disorders,  cardio-metabolic, and musculoskeletal conditions were associated with the recurrence of depression-related work disability among employees who had returned to work after a depression-related disability episode. | 9,946 Finnish public sector employees with at least one depression-related disability episode during 2005–2011 after which the employee had returned to work (14,172 depression-related work disability episodes derived from national health and disability registers for 9,946 individuals) | Without comorbid condition  Other psychiatric disorder  Cardiovascular disease  Hypertension  Diabetes  Musculoskeletal disorder  **Number of comorbidities**  **Covariates:**  Sex  Age  SES  Employment contract | Recurrence of work disability due to depressive disorders.  Results for covariates were not included in paper. | **Model adjusted for covariates and allowing for comorbidities**  **Other psychiatric disorder:** HR=1.76 (1.64;1.88)  **Cardiovascular disease:** HR=1.54 (1.25;1.89)  **Hypertension:** HR=1.46 (1.29;1.66)  **Diabetes:** HR=1.72 (1.49;2.00)  **Musculoskeletal disorder:** HR=1.34 (1.23;1.45)  **One comorbid condition:** HR=1.51 (1.41;1.62)  **Two to four comorbid conditions:** HR=1.73 (1.57;1.90) |
| 6 | Arends et al. [90]  2014  The Netherlands | Prospective study.  Follow-up 12 months.  The aim of this study was to investigate whether sociodemographic, disease-related, personal,  and work-related factors – measured at baseline – are predictors of recurrent sickness absence (SA) at 6 and 12 months follow-up. | 158 participants  (80 participants in the intervention group, 78 in the control group), workers who returned to work after SA due to common mental disorders (CMD). | **Sociodemographic factors**  Gender, Age, Education  Cohabiting  **Disease-related factors**  General health  ≥1 chronic diseases  HADS scores (0–21)  Anxiety  Depression  4DSQ distress score (0–32)  Psychopharmacologic  medication use  Duration of SA  **Personal**  UCL subscale scores (type  of coping)  Problem-focused  Emotional (5–20)  Avoidance (4–16)  **Work-related factors**  Tenure  Contract type  Company size  Supervising role  Monthly income  Accommodation for RTW  Number of OP consultations  RTW percentage  at baseline  WRFQ total score (0–100)  Work engagement (0–6)  RSAW total score (0–24)  **JCQ subscale scores**  Decision latitude  Psychological job demands  Supervisor social support  Colleague social support  Conflicts with colleagues  Conflicts with supervisor  Job insecurity | Recurrent SA at 6 and 12 months follow-up. | **Sociodemographic factors**  Cohabiting  No: OR=1.64 (0.88;3.07); p-value=0.12  **Disease-related factors**  Psychopharmacologic Medication use  Yes: OR=1.78 (0.93;3.40)  p-value=0.08  ≥1 chronic diseases  In multivariate model:  Yes: OR=0.54 (0.30;0.96)  p-value=0.04  **Work-related factors**  Company size  In multivariate model:  ≥100: OR=2.59 (1.40;4.80)  p-value=0.00  RTW percentage at baseline  13–49: OR=1.52 (0.73;3.20)  p-value=0.27  >49: OR=1.77 (0.89;3.51)  p-value=0.10  **JCQ subscale scores**  Supervisor social support  10–12: OR=0.56 (0.29;1.08)  p-value=0.08  >12: OR=0.55 (0.20;1.54)  p-value=0.26  Colleague social support  12: OR=0.60 (0.31;1.17)  p-value=0.14  >12: OR=0.66 (0.28;1.52)  p-value=0.32  Conflicts with supervisor  Sometimes, often or always:  In multivariate model:  OR=2.21 (1.21;4.04); p-value=0.01  Backward elimination for the inclusion of covariates in the model. |
| 7 | Sado et al. [91]  2015  Japan | Retrospective cohort study.  The objective of this study was to investigate which variables could predict repeated sick leave for workers with a history of sick leave because of mental disorders. | 194 subjects employed at a manufacturing company with SA for CMD (disorders listed in the Diagnostic and Statistical Manual of  Mental Disorders, fourth edition (DSM-IV). | **Sociodemographic**  Age at return to work  Sex  Age at initial employment  Tenure (years)  **Diagnostic**  Number of previous sick-leave episodes  **Diagnosis**  Major depressive disorder  Adjustment disorder  Bipolar disorder  Anxiety disorder  Schizophrenia  Others  **Duration of sick leave (days)**  **Work related- Employee rank**  Manager  Senior staff  Normal staff  Assistant staff | Time to repeated sick-leave (duration between the RTW and the repeated sick leave). | **Age at RTW:** HR=0.92 ( 0.87;0.98)  **Sex**: HR=0.59 (0.19;1.77)  **Number of previous sick-leave episodes:** HR=4.86 (2.16;10.94)  Inclusion of variables in the model was based on the forced entry method and the forward selection method. |
| 8 | Endo et al. [92]  2015  Japan | Longitudinal cohort study on the prognosis of RTW in employees with depression.  Follow-up 2 years. | 540 full-time employees at the biggest telecommunication company in Japan who returned to work from April 2002 to March 2008 after their first  sick leave due to depression. | Age  Gender  Duration of the first sickness absence  Living with family/alone  Time for commute  Manager/non-manager  Job title  Brief Job Stress Questionnaire:  Organizational job demand  Organizational job control | Recurrent SA | ***Univariable analysis***  **Age ≥ 42:** HR= 0.878 (0.665–1.160)  **Gender wome**n: HR= 0.875 (0.590–1.297)  **Duration of the first sickness absence ≥130 days:** HR= 0.973 (0.726–1.304)  **Living alone:** HR= 1.341 (0.959–1.847)  **Time for commute (min) >71:** HR= 0.805 (0.603–1.076)  **Manager**: HR= 1.063 (0.725–1.559)  **Job title**  Ofﬁce worker (ref)  Sales worker: HR= 0.734 (0.509–1.060)  Technician: HR= 0.844 (0.599–1.189)  Researcher: HR= 0.622 (0.197–1.964)  **Organizational job demand high:** HR=1.385 (1.04–1.87)  **Organizational job control low:** HR= 1.057 (0.80–1.40)  ***Multivariable analysis:***  **Organizational job demand:** HR 1.46 (1.01–2.10) |
| 9 | Norder et al. [93]  2015  The Netherlands | Cohort study.  Follow-up 10 years. | 14,369 workers (8,164 production and 6,205 office workers) | Age  Gender  Marital status  Employment | Recurrences were defined as any mental SA episode occurring >28 days after recovery from index mental SA | **In production workers and office workers**  Age <25 years: HR=1 (ref)  Age>55 years: HR=3.73 (1.84–5.55)  **Gender men:** HR= 0.85 (0.42–1.73)  **Marital status, being single**  Production workers: HR= 1.14 (0.77–1.70)  Office workers: HR= 1.07 (0.56–2.05)  **Employment part-time**  Production workers: HR= 1.12 (0.46–2.75)  Office workers: HR= 1.02 (0.55–1.89) |
| 10 | Norder et al. [62]  2015  The Netherlands | Cohort study.  Follow-up 10 years.  Study aimed to investigate the risk of recurrent mental health SA. | 5,826 male production workers of a steel plant. | Age  Marital status  Work hours per week  Employment duration  Occupational grade (unskilled, semi-skilled, skilled, technician / supervisor) | Recurrent mental SA was defined as any SA episode occurring >28 days after recovery from the first mental SA episode. | Being a shift worker: HR= 1.04 (0.62–1.74)  The risk of recurrent mental health SA did not differ between shift and day workers. |
| 11 | Real et al. [64]  2016  Spain | Cohort study.  Follow-up 4 years.  The aim of this study  was to gather data on long-term sickness absence (and relapse) due to mental disorders in Spain. | A cohort of 7,112 Spanish patients during the period 2008–2012.  A cohort study of workers registered with a mutual healthcare provider and who had  begun a period of sickness absence due to a mental disorder. | Socio-demographic (age, gender, town/city of residence), clinical (psychiatric diagnosis according to ICD-9), employment-related (type of contract, type of social security contributions, and sector in which employed, coded according to the National Classification of Economic Activities, CNAE 2009), and those related to the sickness absence (duration, reason for end of sick leave). | Recurrent events of sick leave separated by fewer than 180 days (between the last day of  a previous event and the first day of the next) were considered as relapses. | **Gender male**: OR= 1.172 (0.991–1.366)  Type of social security contributions  **General scheme**: OR= 1.178 (0.922–1.508)  **Self-employed**: ref. |
