# Supplementary material for: Determinants of Sickness Absence and Return to Work Among Employees with Common Mental Disorders: A Scoping Review
Source: J Occup Rehabil. 2017 Oct 4;28(3):393–417. doi: 10.1007/s10926-017-9730-1 (PMC6096498; doi:10.1007/s10926-017-9730-1)
Supplement: Supplementary file 2 — Supplementary material 2 (DOCX 18 KB) [file 10926_2017_9730_MOESM2_ESM.docx]

| **Supplemental Table 2:** the relation between different CMD conditions and SA, RTW and recurrent SA. | | |
| --- | --- | --- |
|  | CMD diagnose groups | Descriptive relation between diagnosis and work outcome |
| **Sickness absence** | | |
| Laitinen-Krispijn [25] | Major depression disorder Dysthymia  Anxiety disorders  Substance use disorders  Any mood disorder | In men, major depressive disorder, dysthymia, simple phobia and drug abuse/dependence were associated with an increased likelihood of SA. Anxiety disorders were not associated with SA.  In women, none of the 1-year DSM-III-R disorders was associated with a significantly (p< 0.05) increased likelihood of SA. |
| Buist-Bouwman [29] | Mood disorder  Anxiety disorder  Substance use disorder | Mood disorder was the mental disorder associated with the most excess impairment days (EID) (28.9), followed by anxiety disorder (17.6) and substance use disorder (7.6).  (which means that people with a mood disorder were on average 28.9 days more absent from work than people of the same sex, age and educational level who did not have a mood disorder in the past year). |
| Peterson et al. [42] | Burnout  Depression  Anxiety | Burnout (subscale exhaustion) is the strongest predictor for SA, depression is a stronger predictor than anxiety.  OLBI exhaustion was associated with future longtime SA: OR =5.17 (CI, 3.06 to 8.76); HAD-depression OR=1.12 (CI, 1.02 to 1.22); and HAD-anxiety OR=1.08 (CI, 0.99 to 1.18). |
| Roelen et al. [57] | Distress  Depression  Anxiety  Somatization | The standardized distress, depression, anxiety and somatization scores at baseline were positively associated with future high all-cause SA during follow-up, with somatization and depression as strongest predictors. |
| Hendriks et al. [60] | Depressive disorders  Anxiety disorders | Healthy controls reported the least work disability at all measurement (all p-valueso.001). Subjects with a history of anxiety and/or depressive disorder came next, followed by those with pure anxiety disorders and pure depressive disorders. Those with comorbid anxiety–depressive disorder reported the most work disability at all measurements. Subjects with depressive disorders were signiﬁcantly less disabled at work than those with comorbid anxiety– depressive disorder. |
| Real et al. [64] | Adaptive disorders  Affective disorders  Anxiety disorders | Affective disorders and personality disorders were more commonly associated with long-term sickness absences (followed by psychotic disorders and substance dependence), whereas anxiety disorders were more likely in the case of short-term sick leave. |
| **RTW** | | |
| Nieuwenhuijsen [69] | Depression or anxiety disorders  Adjustment disorders | Having depression or anxiety disorders is a predictors of longer time to return to work (reference group adjustment disorders). |
| Hoedeman et al. [73] | Depressive disorders  Anxiety disorders  Health anxiety  Distress  Somatization | The Cox regression analysis showed that high somatic symptom severity (somatization) and health anxiety, but not psychiatric co-morbidity (depressive, anxiety and panic disorder) contribute to a longer duration of sickness absence. |
| Nielsen et al. [74] | Stress-burnout  Depression  Other mental health problem | Participants sick-listed with self-reported stress/ burnout returned faster to work compared to those sick-listed with self-reported depression (HR=0.73, CI: 0.60–0.88) and with other MHPs (HR=0.46, CI: 0.27–0.78). |
| Virtanen et al. [75] | Adjustment disorders  Anxiety disorders  Depression  Bipolar disorders | Among participants with different psychiatric diagnoses, the most likely to return to work were those with a diagnosis of ‘reaction to severe stress and adjustment disorders’ (93%) followed by participants with anxiety disorders (76%), depressive disorders (63%) and bipolar disorders (54%). Participants with substance-use disorders (36%), schizophrenia and related disorders (41%), and personality disorders (45%) were the least likely to return to work. |
| Soegaard et al. [79] | Depression  Anxiety  Somatoform disorder | The rate of return to work was highest by a rate of 118.5 individuals/1000 sick-listed individuals/30 days for individuals who did not have a verified psychiatric disorder. This was followed by anxiety (101.7), depression (60.8), and somatoform disorder (41.2).  Depression showed a signiﬁcantly lower rate of return to work and anxiety showed a signiﬁcantly higher rate. Somatoform disorder showed a signiﬁcantly higher rate of return to work except for individuals who were white collar/civil servant where the rate was signiﬁcantly lower. |
| Ekberg et al. [82] | Depression  Anxiety  Stress or burnout | There was no major difference in the distribution of diagnoses between returning within 3 months and within 3–12 months: 41 and 48 % respectively had diagnoses of depression, 15 and 14 % respectively suffered from anxiety, 39 and 33 % respectively suffered from stress or burnout according to the medical records. No differences in RTW between the different diagnoses we found. |
| **Recurrent SA** | | |
| Koopmans et al. [86] | Distress  Adjustment disorders  Depressive disorders  Anxiety | Sickness absence due to psychiatric disorders (anxiety and depression) does not have a higher recurrence density of sickness absence due to any CMDs as compared to stress-related disorders (distress and adjustment disorders). |
| Koopmans et al. [88] | Distress symptoms  Depressive symptoms  Adjustment disorder  Anxiety symptoms | In men, depressive symptoms were related to higher recurrent SA due to CMDs than distress symptoms and adjustment disorders. In women, no differences by diagnostic category were found. |
| Sado et al. [91] | Depression  Anxiety disorders  Adjustment disorders  Bipolar disorders | Diagnosis was not a predictor for recurrent SA, no significant differences were observed. |
